# Continuous wound infiltration versus epidural analgesia for midline abdominal incisions – a randomized-controlled pilot trial (Painless-Pilot trial; DRKS Number: DRKS00008023)

**DOI:** 10.1371/journal.pone.0229898

**Published:** 2020-03-06

**Authors:** Rosa Klotz, Svenja E. Seide, Phillip Knebel, Pascal Probst, Thomas Bruckner, Johann Motsch, Alexander Hyhlik-Dürr, Dittmar Böckler, Jan Larmann, Markus K. Diener, Markus A. Weigand, Markus W. Büchler, Andre L. Mihaljevic

**Affiliations:** 1 Department of General, Visceral and Transplantation Surgery, University Hospital Heidelberg, Heidelberg, Germany; 2 Institute of Medical Biometry and Informatics, University of Heidelberg, Heidelberg, Germany; 3 Department of Anaesthesiology, University Hospital Heidelberg, Heidelberg, Germany; 4 Department of Vascular and Endovascular Surgery, University Hospital Heidelberg, Heidelberg, Germany; KU Leuven, BELGIUM

## Abstract

**Objectives:**

To test the feasibility of a randomized controlled study design comparing epidural analgesia (EDA) with continuous wound infiltration (CWI) in respect to postoperative complications and mobility to design a future multicentre randomized controlled trial.

**Design, setting, participants:**

CWI has been developed to address drawbacks of EDA. Previous studies have established the equivalent analgesic potential of CWI compared to EDA. This is a single centre, non-blinded pilot randomized controlled trial at a tertiary surgical centre. Patients undergoing elective non-colorectal surgery via a midline laparotomy were randomized to EDA or CWI. Endpoints included recruitment, feasibility of assessing postoperative mobility with a pedometer and morbidity. No primary endpoint was defined and all analyses were explorative.

**Interventions:**

CWI with local anaesthetics (experimental group) vs. thoracic EDA (control).

**Results:**

Of 846 patients screened within 14 months, 71 were randomized and 62 (31 per group) included in the intention-to-treat analysis. Mobility was assessed in 44 of 62 patients and revealed no differences within the first 3 postoperative days. Overall morbidity did not differ between the two groups (measured via the comprehensive complication index). Median pain scores at rest were comparable between the two groups, while EDA was superior in pain treatment during movement on the first, but not on the second and third postoperative day. Duration of preoperative induction of anaesthesia was shorter with CWI than with EDA. Of 17 serious adverse events, 3 were potentially related to EDA, while none was related to CWI.

**Conclusion:**

This trial confirmed the feasibility of a randomized trial design to compare CWI and EDA regarding morbidity. Improvements in the education and training of team members are necessary to improve recruitment.

**Trial registration:**

DRKS00008023.

## Introduction

Sufficient analgesia is a prerequisite for the successful perioperative management of patients undergoing surgery and reduces postoperative complications and the development of chronic pain [1,2]. In major abdominal surgery, different analgesic techniques such as systemic intravenous patient-controlled analgesia (PCA) or regional techniques, such as epidural analgesia (EDA), are established standard procedures for effective perioperative pain control [3,4]. However, the ideal analgesic technique in abdominal surgery remains unclear and further RCTs are needed [4]. More recently new locoregional analgesic techniques like continuous wound infiltration (CWI), in which a local anaesthetic is continuously applied into the laparotomy wound via an elastomer pump, have been developed as potential alternatives to address drawbacks of EDA and PCA [5].

Specifically, EDA has been criticised for causing rare, but serious adverse events, its multiple contraindications, high failure rates [6], associated high personal and material costs [7] and the associated immobilization patients due to equipment and urinary catheters [4]. Despite these disadvantages, EDA compares favourably to systematic opioid use in some surgical specialties and for high risk patients [8–11]. A number of additional advantages like enhanced pain control, reduced consumption of anaesthetics, reduction of the surgical stress response and early bowel recovery have been postulated for EDA, however overall evidence is sparse [4,12]. Furthermore, depending on trial design, study population, and comparators the effect of EDA on clinically relevant outcomes such as morbidity and mortality are heterogeneous [4,13,14].

CWI could potentially circumvent EDA-associated problems as placement into the laparotomy wound is fast and simple and the technique does not carry the risk of potentially detrimental epidural hematoma or infection. Also, CWI has already been shown to successfully treat postoperative pain and a number of trials have established the equivalent analgesic potential of CWI vs. EDA following abdominal surgery [5,15].

Until now, however, no study has compared the overall postoperative morbidity of CWI versus EDA directly. This is important, as postoperative morbidity and mortality in conjunction with pain control are arguably the most patient-relevant outcomes. Also, the widespread implementation of EDA has been justified by its supporters because of its potential beneficial effect on postoperative recovery and pain [16]. Therefore, superiority in terms of postoperative complications in conjunction with sufficient (non-inferior) pain control, could establish CWI as a therapeutic alternative for patients undergoing major abdominal surgery.

Therefore, our current study was designed as a randomized-controlled pilot trial (RCT) comparing EDA vs. CWI in patients undergoing major upper gastrointestinal surgery with the focus on assessing feasibility of recruitment and feasibility of assessing postoperative patient mobility via a pedometer Immobility has been associated with adverse outcomes like pulmonary and thrombotic complications and is discouraged in all current enhanced recovery from surgery (ERAS) guidelines [17]. Therefore, the objective unbiased assessment of postoperative mobility is an important aspect of future RCTs. Finally, we aimed to raise preliminary data on postoperative complications and pain to plan and conduct a future large multicentre RCT.

## Methods

The Painless-Pilot trial was a randomized, controlled, non-blinded, single centre pilot trial with two parallel study groups performed at the Department of General, Visceral and Transplantation Surgery at Heidelberg University Hospital. The trial was performed according to the IDEAL recommendations (step 2b exploration) [18] and the results are reported according to current CONSORT guidelines with the extension for pilot and feasibility trials [19] (see S1 Checklist). In accordance with the professional code for physicians in Germany (§15 BOÄ), the study protocol has been approved by the local independent ethics committee of the medical faculty of the University of Heidelberg (S-231/2015) on 11th August 2015 and was conducted in accordance with the Declaration of Helsinki in its current version. It was registered at the German Clinical Trials Register (DRKS00008023) and no changes to the methods or the design of the study occurred after registration. Registration of the study was applied for in April 2015. All queries from the DRKS were answered until the 31^st^ August 2015 except the planned inclusion date of the first patient (first-patient-in), which was correct in the DRKS registry on 1^st^ December 2015. Confirmation of registration occurred on 4^th^ December 2015. The first patient was recruited and randomized into the study on 20^th^ October 2015. Until 4^th^ December 2015 eight patients were randomized into the trial. The authors confirm that all ongoing and related trials for this drug/intervention are registered.

### Inclusion/exclusion criteria

All patients aged 18 years or more scheduled for elective upper abdominal surgery (including upper gastrointestinal, pancreatic, hepatobiliary, vascular and other surgery) via a midline laparotomy with an indication for EDA were screened for eligibility. Rectal surgeries were excluded, because of the frequent need for stoma placement, which creates an abdominal incision not reached by the CWI catheter. Furthermore, patients with chronic pain and/or regular consumption of opioid analgesics were excluded due to potential confounding. Patients unable to walk unaided were not admitted as postoperative mobility could not be evaluated in these patients. Further exclusion criteria were a contraindication for EDA or CWI, planned stoma placement, pregnancy or breast-feeding and participation in another interventional trial with interference with the intervention or endpoint of this trial. Only patients able to understand the character and all relevant aspects of the trial including aims, methods, risks and individual consequences of this trial who provided written informed consent were included.

### Randomisation and blinding

In order to achieve comparable intervention groups for known and unknown risk factors, patients were allocated randomly to the two treatment groups using randomizer.at, a web-based tool from university Graz (Randomizer, Medical University of Graz, Institute for Medical Informatics, Statistics and Documentation (IMI)). A block-randomization with a block length of four was implemented. Randomization was performed block wise (permuted blocks) the day before or at the day of surgery at the Study Centre of the Department of General, Visceral, and Transplantation Surgery by the responsible study nurse. Blinding of patients, anaesthesiologists and outcome assessors to the intervention was not implemented as the insertion of the epidural catheter is performed when the patient is awake. Moreover, the difference between CWI and EDA will be obvious to patients and observers postoperatively. Given the pilot aspect of this trial and the objective nature of most endpoints a non-blinded design of the trial seemed justifiable.

### Trial intervention and control group

Patients in the intervention group received a CWI catheter, which was placed suprafascially in the surgical site of the midline laparotomy at the end of surgery after closure of the abdominal fascia and before closure of the subcutaneous tissue and skin. The catheter covered the complete length of the incision (S1 and S2 Figs). If necessary, more than one catheter was used. A CWI catheter (On-Q® PainBuster®, B.Braun, Melsungen, Germany) with Ropivacain 0.2% isobar at 5ml/h for 3 days connected to an elastomeric pump continuously releasing the local anaesthetic was used.

In the control group patients received a thoracic EDA (Perifix®Komplett, Fa. B.Braun Melsungen) during preoperative induction of anaesthesia at Th 7/8 for upper GI procedures and hepatobiliary surgery or Th 8/9 for pancreatic surgery according to internal standard operating procedures. The catheter was inserted 5–6 cm into the epidural space. 5 mL Lidocain 2% were injected as a testing dose to exclude intrathecal placement. Before surgical incision, 10mL Ropivacaine 0.5% supplemented with 4mL Sufenta (R) epidural (20μg sufentanil) were injected as a loading dose to facilitate epidural analgesia. To maintain epidural analgesia during surgery a continuous infusion of 8 mL/h 0.2% Ropivacain was established until skin closure. In the postoperative period EDA was achieved with ropivacain 0.2% with sufentanil at 6-10ml/h administered for 3 days according to the following scheme: on postoperative day 1 0.5μg/ml were administered, on day 2 the sufentanil dose was reduced to 0.25μg/ml. From day 3 on 0.2% ropivacaine was given without additional sufentanil.

All patients were seen by a dedicated acute pain service on a daily basis. A physician and a pain nurse evaluate the puncture side for signs of infection or catheter dislocation. During every visit pain level at rest and during movement were assessed and cold sensations were tested to confirm sufficient pain control. In addition, all patients were screened for neurologic deficits including assessment of muscle tone and strength.

### Perioperative standards

Abdominal wound closure was performed in a standardized manner in small-stitches technique^29^ via a running suture with Monomax® (Fa. B. Braun Melsungen, Germany). No subcutaneous drains were placed in both groups with the exception of the CWI in the intervention group. Overall postoperative management was performed identically in both intervention groups according to hospital standard.

Both, CWI and EDA catheters were set to be removed at the third postoperative day. In both groups, patients received a standardized additional pain medication with fixed-dose 1g metamizole four times daily, and intravenous oxycodone 5mg up every 4 hours on demand. In case of treatment failure defined as uncontrollable pain with a numeric rating scale of ≥5 despite the above-mentioned treatment regime a systemic oxycodon (1 mG/mL) PCA with no baseline rate, 1.5 mG oxcycodon bolus given over 3 minutes and a 20-minute hold-off period was initiated. Other analgesic medication was not routinely given.

### Outcome measures and assessment

The outcome assessors were study nurses and physicians of the clinical study center of the Department of Surgery. Pain was evaluated by our interprofessional acute-pain team. All outcome assessors were independent of the research team and were well trained before the start of the study. The following postoperative outcome measures were assessed and no changes to the outcome measures occurred after commencement of the trial:

*A*. *Trial feasibility outcome measures*
  1 Accomplished recruitment of n = 70 patients within 6 months
  2 Feasibility of assessing the postoperative mobility of patients via a digital pedometer (OMRON Walking style Pro 2.0, OMRON Medizintechnik Handelsgesellschaft mbH Mannheim, Germany) placed at patients’ right hip bone using adhesive bandage at the end of surgery in at least two thirds of all patients. During the first three postoperative days distance (in meters) and the number of steps were measured.
*B*. *Analgesia-associated outcome measures*
  3 Pain scores at rest and during movement recorded on postoperative day 1–3 via a numeric rating scale (NRS).
  4 Amount of systemic opioid consumption in both groups within the first 3 postoperative days in morphine equivalents. In order to compare different opioids, the following rules were applied: a.) 1 mg oxycodone i.v. equals 1.3 mg morphine i.v., b.) 1 mg oxycodone oral equals 0.5mg morphine i.v., c.) For the conversion of epidural opioids to i.v. opioids we assumed that 1 mg epidural sufentanil equals 1 mg sufentanil i.v. which in turn equals 0.001 mg morphine i.v. based on the studies by Miguel et al. [20] and Taverne et al. [21]. Calculation of opioid consumption in EDA is controversial and some authors have demonstrated a clinical advantage of epidural infusion over i.v. infusion with lower doses needed via epidural application [22]. However, the latter effect is not relevant after the first few postoperative hours [20,22]. For continuous EPA (≥48 hours) as used in our study conversion of epidural doses to i.v. doses in a 1:1 ratio seems justified [23].
  5 Treatment-failure rate defined as uncontrollable pain with a numeric rating scale of ≥5 despite the randomized treatment regime with the need for an i.v. PCA (see above).
  6 Duration of EDA /CWI therapy (in days).
  7 Length of preoperative induction of anaesthesia and length of surgery (in minutes).
  8 Length of hospital stay (in days)
  9 Time to first flatus/bowel movement.
*C*. *Morbidity endpoints*
  10 The overall postoperative morbidity of patients in the study was evaluated based on the comprehensive complication index (CCI) [24,25].
*D*. *Safety parameters*
  11 Frequency and severity of serious adverse events (SAE).
  12 Rate of catheter-related complications in both groups. In the EDA group the rate of catheter infections, dislodgement, neuroaxial hematoma, bleeding into the vertebral canal confirmed by MRI and/or CT scan was assessed. In the CWI group, the rate of surgical side infection (SSI) according to the definition of the Centre for Disease Control and Prevention was applied [26].

### Description of trial visits

Demographic and baseline data were documented during visit 1. Patients were randomized the day before or at the day of surgery (visit 2). Intraoperative parameters were recorded during visit 2. Patients were planned for clinical follow-up at postoperative day 1, 2, 3, 7 and 30–35 (visit 3–7) for evaluation of postoperative outcome measures and SAEs. Items assessed during visits are listed in Table 1.

**Table 1:** Trial visits and documented parameters of the PAINLESS Pilot trial.

Visit	1	2	3–5	6–7
	Screening/Consent	Randomization/Intervention	Post-op day 1,2,3	Post-op day 7 and 30–35
Inclusion/exclusion criteria	**X**			
Informed consent	**X**			
Demographic data/ Medical history	**X**			
Randomization		**X**		
Surgery		**X**		
Intraoperative parameters		**X**		
Number of steps/distance covered			**X**	
Pain scores (NRS)			**X**	
Time to first flatus/bowel movement			**X**	**X**
Body weight			**X**	**X**
Postoperative complications			**X**	**X**
Length of hospital stay				**X**
SAEs			**X**	**X**

NRS: numeric rating scale. SAE: severe adverse event.

### Statistical analyses

No formal sample size calculation was performed as this was a pilot trial. However, 60 patients (30 patients in each group) were deemed necessary to draw valid conclusions about recruitment rates, to achieve preliminary pedometer data and to confirm an expected median CCI of approximately 20 in our mixed patient cohort, as has been reported previously in patients undergoing pancreaticoduodenectomy (20.9 (0–29.6)) [25,27]. Following randomization, a dropout rate of 10 patients (5 in each study arm) was expected leading to an overall recruitment number of 70 patients.

As this was a pilot trial without confirmative character, no primary endpoint was defined and all analyses were strictly explorative. We describe our data as median values along with minimum and maximum values for ordinal or continuous, and relative and absolute frequencies for categorical endpoints, stratified for treatment groups. We used nonparametric estimation methods as the outcomes are either ordinal distributed or, in case of continuous outcomes, because our pilot trial has a small sample size and normality cannot be assumed. We further report Wilcoxon-Mann-Whitney odds (WMWodds) and their confidence intervals as discussed by Franklin Dexter as an effect estimator [28]. All analyses were done using SAS version 9.4.

### Patient and public involvement

Patients and the public were not involved in the development of the protocol.

## Results

### Recruitment

**Fig 1:**
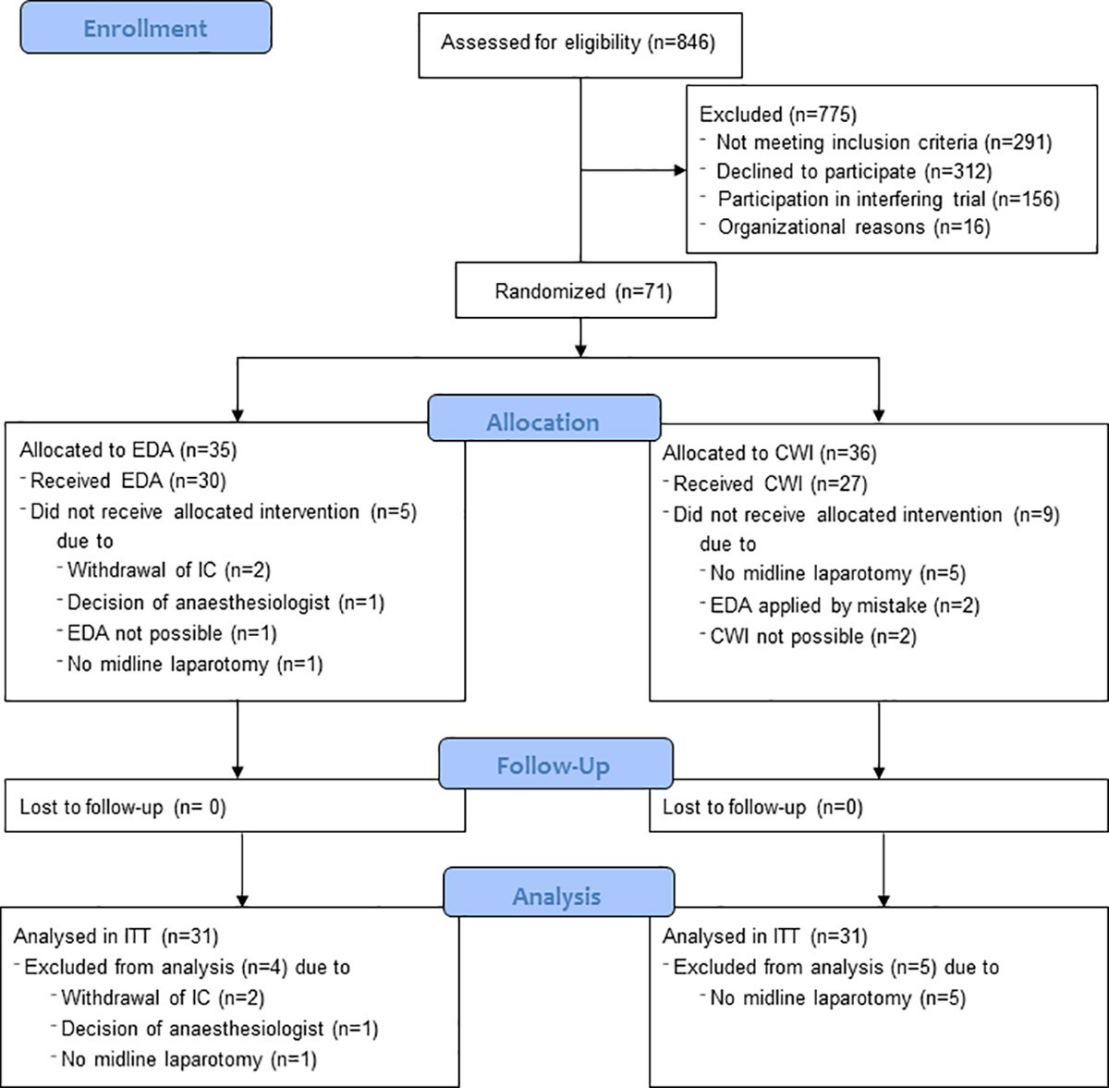
CONSORT flow-chart.

All consecutive patients planned to undergo surgery at the department of General, Visceral and Transplantation Surgery at Heidelberg University Hospital were screened for eligibility. Between Oct 20^th^, 2015 to Dec 15^th^, 2016 (14 months) 846 patients were screened for eligibility of which 71 patients were randomly assigned to CWI (n = 36) or EDA (n = 35) (Fig 1). A total of 9 patients had to be excluded from the pilot trial after randomization either because no median laparotomy was performed (L-incision (n = 2), laparoscopy (n = 4)) or no analgesia via CWI or EDA was indicated because of short operation time (n = 1) or informed consent was withdrawn (n = 2). These patients spread among the two groups as presented in S2 Table. From these patients no data on visits after the surgical procedure was acquired and in particular, no pedometer data was sampled for analysis. Thus, the population of this pilot trial consisted of 71 patients, out of which outcomes could be measured for 62 patients (31 patients in each group).

### Patient characteristics

Baseline characters revealed no difference between the two study groups (Table 2). More males (68.3%) than females were included in the study with an average age of 61.6 +/-12.4 years. Most patients had mild or severe systemic comorbidities indicated by the American Society of Anesthesiologists physical status classification (ASA score) II (66.1%) and III (32.3%). More specifically, 17.7% of trial participants had cardiac and 22.6% had pulmonary comorbidities. The most frequent indications for surgery were pancreatic (56.5%) and upper gastrointestinal diseases (22.6%). One patient planned for upper abdominal surgery, eventually received colorectal surgery, but was not excluded from the ITT analysis.

**Table 2:** Baseline characteristics.

	EDA	CWI	Total
	N = 31	N = 31	N = 62
**Gender**			
• male	21 (67.7%)	21 (67.7%)	42 (67.7%)
• female	10 (32.3%)	10 (32.3%)	20 (32.3%)
**Age** [years] Mean +/- SD	61.6 +/-13.6	61.6 +/-11.4	61.6 +/-12.4
**BMI** [kg/m^2^] Mean +/- SD	25.6 +/-5.3	25.6 +/-4.1	25.6 +/-4.7
**ASA status**			
• I	1 (3.2%)	0 (0.0%)	1 (1.6%)
• II	21 (67.7%)	20 (64.5%)	41 (66.1%)
• III	9 (29.0%)	11 (35.5%)	20 (32.3%)
**Underlying disease**			
• colorectal	0 (0.0%)	1 (3.2%)	1 (1.6%)
• hepatobiliary	0 (0.0%)	1 (3.2%)	1 (1.6%)
• pancreatic	20 (64.5%)	15 (48.4%)	35 (56.5%)
• upper GI	7 (22.6%)	7 (22.6%)	14 (22.6%)
• other	2 (6.5%)	4 (12.9%)	6 (9.7%)
**Cardiac comorbidity**	5 (16.1%)	6 (19.4%)	11 (17.7%)
**Pulmonary comorbidity**	7 (22.6%)	7 (22.6%)	14 (22.6%)
**Type of surgery**			
• colorectal	0 (0%)	1 (3.2%)	1 (1.6%)
• upper gastrointestinal surgery	4 (12.9%)	5 (16.1%)	9 (14.5%)
• pancreatic surgery	17 (54.8%)	13 (41.9%)	30 (48.4%)
• hepatobiliary surgery	1 (3.2%)	1 (3.2%)	2 (3.2%)
• multivisceral resection	2 (6.5%)	3 (9.7%)	5 (8.1%)
• vascular surgery	2 (6.5%)	3 (9.7%)	5 (8.1%)
• other abdominal surgery	2 (6.5%)	1 (3.2%)	3 (4.8%)
• explorative laparotomy	3 (9.7%)	4 (12.9%)	7 (11.3%)
**Insertion of surgical drains**	24 (77.4%)	24 (77.4%)	48 (77.4%)

### Trial feasibility outcome measures

As pointed out above, 14 months were required to include the planned number of 70 patients (5 patients/ month). Thus, our goal of recruiting 70 patients in 6 months (11.7 patients/ month) was not met. However, the second feasibility parameter (measurement of postoperative mobilization via a pedometer in ≥ 2/3 of patients) was successfully accomplished as 44 patients out of 62 in the ITT group received pedometers and data read-out. Reasons for failure were (n = 4 pedometer lost, n = 7 unsuccessful data recording, n = 7 no pedometer attached after surgery). Steps and distance covered by patients in the first postoperative days did not differ between groups in explorative analysis (Table 3). Number of steps increased daily from the first postoperative day until the third postoperative day in both groups (Table 3).

**Table 3:** Postoperative outcomes (median minimum;maximum) and Wilcoxon-Mann-Whitney odds along with their confidence intervals.

	EDA (n = 31)	CWI (n = 31)	WMW odds [lower; upper]
**Pain scores at rest (NRS)**			
• POD 1	1.0 +/-1.3	1.2 +/-1.5	1.14 [0.64; 2.07]
• POD 2	0.7 +/-1.1	1.0 +/-1.5	1.25 [0.74; 2.19]
• POD 3	0.4 +/-0.8	0.7 +/-1.3	1.32 [0.83; 2.16]
**Pain scores at movement (NRS)**			
• POD 1	2.7 +/-1.9	4.4 +/-2.3	2.53 [1.41; 5.59]
• POD 2	2.6 +/-1.8	3.1 +/-2.5	1.20 [0.67; 2.22]
• POD 3	1.6 +/-1.7	1.7 +/-1.7	1.06 [0.59; 1.89]
**Duration of induction of anaesthesia (in min.)**	59.8 +/-13.4	42.9 +/-11.3	5.08 [2.77; 14.66]
**Duration of surgery (in min.)**	218.6 +/-92.8	238.8 +/-116.5	1.30 [0.66; 2.25]
**Duration of catheter therapy (in days)**	3.6 +/-2.4	1.8 +/-1.3	2.95 [1.57; 7.46]
**Time to first bowel movement (in days)**	2.6 +/-0.8	2.8 +/-1.0	1.14 [0.66; 1.99]
**Comprehensive complication index**	21.5 +/- 16.2	18.2 +/- 15.2	1.31 [0.73; 2.47]
**Number of steps POD 1 to POD 3**	979.4+/-1489.7	874.5 +/-1574.5	1.43 [0.71; 3.20]
**Length of hospital stay (in days)**	11.0 +/-7.6	9.6 +/-4.7	1.10 [0.60; 2.02]

CCI: comprehensive complication index. CWI: continuous wound infiltration. EDA: epidural analgesia. NRS: numeric rating scale. POD: postoperative day. WMW odds: Wilcoxon-Mann-Whitney odds

### Analgesia-associated outcome measures

Explorative analysis of median pain scores at rest during the first three postoperative days according to the NRS revealed no differences between the two groups (Table 3). Median (min;max) pain scores during movement were lower with EDA than with CWI only on the first postoperative day, but were not different on postoperative days two or three (Table 3).

The total amount of opioid consumption (including epidural sufentanil application in morphine equivalents) within the first three postoperative days was lower on exploratory analysis in the CWI group than in the EDA group who received a continuous infusion of ropivacaine plus sufentanil (median(min; max) EDA: 216 (15; 429) mg vs. CWI: 102 (0; 318) mg; Wilcoxon-Mann-Whitney odds (WMWodds): 3.35 CI [1.85; 8.19]).

Treatment failure, defined as uncontrollable pain despite the randomized treatment regime with a numeric rating scale of ≥5 with the need of instalment of an i.v. PCA, occurred in 3 patients in the EDA group and 4 patients in the CWI group. Additional PCAs could be implemented on the discretion of the treating physician. Therefore, the total number of PCA placements was high in both groups: seven out of 31 patients in the EDA group received a PCA (23%). Reasons were pain (n = 3), failure of EDA insertion (n = 3) or catheter dislocation (n = 1). Out of 31 patients in the CWI group, 22 received an i.v. PCA (71%) because of pain (n = 8), leakage of local anaesthetic from the wound (n = 1), failed CWI insertion (n = 2) or following relaparotomy (n = 1). An additional 10 patients received a “prophylactic” PCA in the recovery room on the day of surgery, when recovery room physicians not acquainted with the study protocol, assumed potential future inadequate pain control by the CWI, despite patients reporting adequate pain scores in the recovery room with CWI alone.

Explorative analysis revealed that pain-catheter therapy was significantly longer with EDA than with CWI median (min; max) EDA: 4 (0; 9) days versus CWI: 2 (0; 5) days, WMWodds: 2.95 [1.57;7.46]). Catheters were removed prematurely (before postoperative day 3 (POD)) in 11 and 4 cases in the CWI and EDA group, respectively.

Duration of preoperative induction of anaesthesia was shorter (about 17 minutes) with CWI compared to EDA, which were placed during induction of anaesthesia (Table 3). However, median (min;max) duration of surgery did not differ between the two groups (Table 3), although CWIs were placed during surgery, directly after closure of the fascia. Similarly, duration of hospital stay and time to first bowel movement did not differ between both groups (Table 3).

### Morbidity endpoints

Explorative analysis of the CCI revealed no difference between groups (EDA 21 (0; 60.8) versus CWI 15 (0; 59) WMWodds: 1.31 [0.73; 2.47]) with an overall CCI of 19.8 +/-15.7 and single scores between 0 and 60.8. Overall, 13 major complications (defined as Clavien Dindo >II) occurred in 10 patients (EDA: 6, CWI: 7).

### Safety analysis

Safety analyses in terms of SAEs did not show any difference between groups. Overall six patients experienced 17 SAEs with overall ten SAEs in the EDA group and seven SAEs in the CWI group. Three SAEs in patients with an EDA were classified as potentially related to the intervention (fall due to hypotension with serious face injuries, urosepsis associated with prolonged indwelling urinary catheter use and transient neurologic deficit of the lower limb). No SAE was associated with CWI use.

Regarding catheter-related complications, three patients in the EDA group suffered from paraesthesia, one had focal motor deficits, one had a superficial hematoma at the puncture side, one had pain at the puncture side after multiple punctures and one suffered from hypotension. No catheter related complications were recorded for the CWI group. Only one superficial surgical site infection occurred in the EDA group and none in the CWI group.

## Discussion

The main objectives of our pilot study were to inform the decision whether or not to conduct a confirmatory trial comparing EDA with CWI in a multicenter setting and to inform the design of such a potential trial. To this end, 70 patients undergoing elective open upper gastrointestinal surgery were randomly assigned to epidural analgesia (EDA) or continuous wound infiltration (CWI) in the Painless Pilot trial and underwent a broad set of exploratory outcome assessments including trial feasibility-, safety-, morbidity and analgesia-associated endpoints. Recruitment took longer than expected (14 months instead of 6 months). Postoperative mobility was successfully measured via pedometers and showed no difference between the two groups on exploratory analysis. Similarly, morbidity assessment via the comprehensive complication index, pain and safety assessment were successfully established in the study.

Valuable information can be drawn from the results of the PAINLESS Pilot trial for a multicentre RCT comparing EDA vs. CWI following upper gastrointestinal surgery, thus confirming the importance of pilot trials for the planning of high-quality trials in surgery [29]. While the analgesic potential of CWI has been established to be comparable to EDA [5], no high-quality study has yet compared the two methods in regard to their effect on postoperative morbidity. While on one hand the overall results warrant a larger multicentre trial, on the other hand we encountered several problems that would need to be addressed in future studies.

First, recruitment was difficult in our trial and only 71 out of 555 eligible patients were eventually randomized during a 14-month period. This is less than has been reported in other RCTs [5,15,30]. Reasons might partially reflect specificities of our centre as 156 patients were excluded because of interfering trial participation. However, most patients (n = 312) declined to participate. In our trial patient recruitment was hampered in the multidisciplinary teams as not all healthcare professionals were convinced of the clinical equipoise of the two interventions with some favouring CWI and other EDA due to individual opinion. Hence, preoperatively patients repeatedly received diverging information resulting in a lack of informed consent. As analgesia touches multiple stakeholders (patients, anaesthetists, surgeons, nurses, pain teams etc) sufficient training and education of all team members seems vital for the successful conduct of future studies.

Second, based on the preliminary data from our pilot trial a sample size of roughly 712 patients would be necessary to detect the observed mean difference of CCI between EDA and CWI when applying a two-sided t-test with a power of 80% and chosen level of significance of 5% (two-Sample t-tests, calculated with PASS). Consequently, in a future multicenter trial 10 sites could recruit the necessary number of patients in around 15 months.

Third, lack of sufficient information and training likely explains two other problems we encountered during our pilot study, namely the high rate of “prophylactic” PCA placements in the recovery room in the CWI group and the loss of pedometers in about one third of patients. “Prophylactic” placement occurred when recovery room physicians not acquainted with the study protocol, assumed potential future inadequate pain control by the CWI, despite patients reporting adequate pain scores in the recovery room with CWI alone. Similarly, pedometers were frequently removed by nursing personnel not acquainted with the specifics of our study. Pedometer bracelets, rather than attaching the pedometer to the patients’ hip with adhesive tape, might circumvent this problem in future trials.

Fourth, as in previous trials CWI was not inferior to EDA in pain control on explorative analysis [5] with exception of pain during movement on the first postoperative day. The latter seems of minor clinical relevance, as a difference of less than two points on the numeric rating scale is considered to be clinically irrelevant in pain research and salvage application of analgesia is easily possible. Similar to our results, a recent RCT comparing EDA vs. CWI in colorectal surgery has reported only a transient superior analgesic effect of EDA on the first postoperative day [31]. However, interpretation of pain data in our trial is hindered by the high rate of PCA use in the EDA and especially the CWI group. The PCA rate in the EDA group was higher than generally reported in the literature [32] and might be due to previous overestimation of the reliability of the EDA [33,34] and might partially explain previous results that have shown inadequate postoperative pain management [13]. Given that the total amount of opioid consumption was lower in the CWI group, the high rate of PCA placement in the CWI group cannot be explained by inadequate pain control. As pointed out above some PCAs were placed prophylactically in the recovery room, an issue which could be addressed by improved training of study personnel. Finally, gender differences between the two groups might account for differences in pain perception [35] and future trials should therefore implement stratification according to sex.

Fifth, several CWI catheters were also removed early (and PCA placed) because of leakage of local anaesthetics from the wound. This problem was caused by the subcutaneous placement of the catheter, the relatively high flow rate of the CWI in our study compared to previous trials [5,15] and the lack of subcutaneous fat in cachectic tumour patients. Hence in future trials the catheter position might be change to a preperitoneal position. At the time we planned our trial, the optimal catheter position for CWIs in abdominal surgery was unclear and available results indicated that the catheter position has no effect on analgesic effect [5]. In the meantime a meta-analysis indicates that preperitoneal catheter positions for CWI are superior to subcutaneous positions in terms of pain control [15]. Thus, a preperitoneal position seems warranted in future trials.

### Strength and limitations

Our pilot study established the feasibility of measuring the CCI [24,25] in a randomized trial design comparing EDA and CWI. Our preliminary data confirms CCI scores in comparable patient populations [25,36]. The CCI seems a suitable endpoint for comparing postoperative complications as it catches the entirety of complications, rather than focusing on a single complication as has been done in previous trials comparing analgesic techniques [8,37]. As the CCI is based on the established Dindo-Clavien classification of complications, its evaluation is objective and not influenced by an unblinded trial design. Finally, pedometers seem a promising tool for evaluating postoperative mobility. They allow a standardized and objective readout and might circumvent problems associated with mobility scores or questionnaires. Early mobilization is a crucial component of the enhanced recovery after surgery (ERAS) clinical care protocol [17] and a fundamental for prophylaxis of deep vein thrombosis and pulmonary embolism [38].

A limitation of our trial was the unblinded trial design. Blinding EDA and CWI procedures would require significant efforts. EDA catheters are placed when the patient is awake making blinding impractical. A potential way of blinding would be to place sham EDA or CWI catheters. However, this not only raises ethical concerns, but also contradicts the pragmatic nature of the trial as it would create an artificial intervention with two catheters in each patient. Furthermore, it is still unclear if such blinding measures really reduce performance and detection bias [39]. Although we tried to compensate for a potential reporting bias by choosing objective outcomes measures like the CCI or pedometer data, we cannot rule out detection bias for more subjective outcome measures like pain.

### Safety

It was crucial to establish that the CWI does not inflict any potential harm on patients. Although postoperative overall morbidity in the CWI group evaluated via the CCI, SAEs, rate of catheter related complications and surgical side infections were not different in the two groups, the rate of SAEs in the EDA group was high. This is in line with recent reports that have shed a more critical light on EDA-associated adverse events [13,40].

As expected, duration of induction of anaesthesia was shorter with CWI in our trial, which is a relevant economic advantage for our health care system compared to EDA. This supports recent studies that have outlined the high costs associated with EDA [7].

## Conclusion

Our findings support the increasing recognition of CWI as a useful and safe technique within a multimodal approach to postoperative pain management [15]. Based on results of our pilot study, a large multicentre RCT comparing CWI and EDA in terms of postoperative complications, pain and mobility seems feasible. As indicated above, several modifications to the trial design are necessary to address the problems identified in our pilot study.

## Supporting information

S1 Checklist CONSORT check-list. (DOC)

S1 Fig Placement of the continuous wound infiltration catheter in the median laparotomy wound after closure of the fascia. Reprinted with permission from Mann V. et al. Chirurg. 2011 Oct;82(10):906–12.
(TIF)

S2 Fig Continuous wound infiltration catheter after skin closure. (TIF)

S1 Table Individual patient data. (CSV)

S2 Table Reasons for exclusion from ITT set per treatment group. (DOCX)

S1 Protocol (DOC)

## References

[1] Perkins FM, Kehlet H. Chronic pain as an outcome of surgery. A review of predictive factors. Anesthesiology. 2000;93: 1123–1133. 10.1097/00000542-200010000-00038 11020770

[2] Kehlet H, Wilmore DW. Multimodal strategies to improve surgical outcome. Am J Surg. 2002;183: 630–641. 10.1016/s0002-9610(02)00866-8 12095591

[3] Laubenthal H, Neugebauer E. S3-Leitlinie Behandlung akuter perioperativer und posttraumatischer Schmerzen. Deutsche Interdisziplinäre Vereinigung für Schmerztherapie (DIVS) e.V; 2009. 10.1055/s-2006-949507 16874569

[4] Salicath JH, Yeoh EC, Bennett MH. Epidural analgesia versus patient-controlled intravenous analgesia for pain following intra-abdominal surgery in adults. Cochrane Database Syst Rev. 2018;8: CD010434 10.1002/14651858.CD010434.pub2 30161292 PMC6513588

[5] Ventham NT, Hughes M, O’Neill S, Johns N, Brady RR, Wigmore SJ. Systematic review and meta-analysis of continuous local anaesthetic wound infiltration versus epidural analgesia for postoperative pain following abdominal surgery. Br J Surg. 2013;100: 1280–1289. 10.1002/bjs.9204 24244968

[6] Duncan F. Prospective observational study of postoperative epidural analgesia for major abdominal surgery. J Clin Nurs. 2011;20: 1870–1879. 10.1111/j.1365-2702.2011.03752.x 21615577

[7] Babazade R, Saasouh W, Naylor AJ, Makarova N, Udeh CI, Turan A, et al The cost-effectiveness of epidural, patient-controlled intravenous opioid analgesia, or transversus abdominis plane infiltration with liposomal bupivacaine for postoperative pain management. J Clin Anesth. 2018;53: 56–63. 10.1016/j.jclinane.2018.10.003 30326379

[8] Liu SS, Wu CL. Effect of postoperative analgesia on major postoperative complications: a systematic update of the evidence. Anesth Analg. 2007;104: 689–702. 10.1213/01.ane.0000255040.71600.41 17312231

[9] Bardia A, Sood A, Mahmood F, Orhurhu V, Mueller A, Montealegre-Gallegos M, et al Combined Epidural-General Anesthesia vs General Anesthesia Alone for Elective Abdominal Aortic Aneurysm Repair. JAMA Surg. 2016;151: 1116–1123. 10.1001/jamasurg.2016.2733 27603002

[10] Pöpping DM, Elia N, Van Aken HK, Marret E, Schug SA, Kranke P, et al Impact of epidural analgesia on mortality and morbidity after surgery: systematic review and meta-analysis of randomized controlled trials. Ann Surg. 2014;259: 1056–1067. 10.1097/SLA.0000000000000237 24096762

[11] Rodgers A, Walker N, Schug S, McKee A, Kehlet H, van Zundert A, et al Reduction of postoperative mortality and morbidity with epidural or spinal anaesthesia: results from overview of randomised trials. BMJ. 2000;321: 1493 10.1136/bmj.321.7275.1493 11118174 PMC27550

[12] Panousis P, Heller AR, Koch T, Litz RJ. Epidural ropivacaine concentrations for intraoperative analgesia during major upper abdominal surgery: a prospective, randomized, double-blinded, placebo-controlled study. Anesth Analg. 2009;108: 1971–1976. 10.1213/ane.0b013e3181a2a301 19448234

[13] Kooij FO, Schlack WS, Preckel B, Hollmann MW. Does regional analgesia for major surgery improve outcome? Focus on epidural analgesia. Anesth Analg. 2014;119: 740–744. 10.1213/ANE.0000000000000245 25137006

[14] Revie EJ, McKeown DW, Wilson JA, Garden OJ, Wigmore SJ. Randomized clinical trial of local infiltration plus patient-controlled opiate analgesia vs. epidural analgesia following liver resection surgery. HPB (Oxford). 2012;14: 611–618. 10.1111/j.1477-2574.2012.00490.x 22882198 PMC3461387

[15] Mungroop TH, Bond MJ, Lirk P, Busch OR, Hollmann MW, Veelo DP, et al Preperitoneal or Subcutaneous Wound Catheters as Alternative for Epidural Analgesia in Abdominal Surgery: A Systematic Review and Meta-analysis. Ann Surg. 2018 10.1097/SLA.0000000000002817 29781846

[16] Weiss R, Pöpping DM. Is epidural analgesia still a viable option for enhanced recovery after abdominal surgery. Curr Opin Anaesthesiol. 2018;31: 622–629. 10.1097/ACO.0000000000000640 29994937

[17] ERAS Society Guidelines. In: Eras [Internet]. [cited 4 Dec 2018]. Available: http://erassociety.org/guidelines/list-of-guidelines/

[18] McCulloch P, Altman DG, Campbell WB, Flum DR, Glasziou P, Marshall JC, et al No surgical innovation without evaluation: the IDEAL recommendations. Lancet. 2009;374: 1105–1112. 10.1016/S0140-6736(09)61116-8 19782876

[19] Schulz KF, Altman DG, Moher D. CONSORT 2010 Statement: Updated Guidelines for Reporting Parallel Group Randomized Trials. Ann Intern Med. 2010;152: 726–732. 10.7326/0003-4819-152-11-201006010-00232 20335313

[20] Miguel R, Barlow I, Morrell M, Scharf J, Sanusi D, Fu E. A prospective, randomized, double-blind comparison of epidural and intravenous sufentanil infusions. Anesthesiology. 1994;81: 346–352; discussion 25A-26A. 10.1097/00000542-199408000-00012 8053584

[21] Taverne RHT, Ionescu TI, Nuyten STM. Comparative Absorption and Distribution Pharmacokinetics of Intravenous and Epidural Sufentanil for Major Abdominal Surgery. Clin Pharmacokinet. 1992;23: 231–237. 10.2165/00003088-199223030-00005 1387351

[22] Geller E, Chrubasik J, Graf R, Chrubasik S, Schulte-Mönting J. A randomized double-blind comparison of epidural sufentanil versus intravenous sufentanil or epidural fentanyl analgesia after major abdominal surgery. Anesth Analg. 1993;76: 1243–1250. 10.1213/00000539-199306000-00011 8498661

[23] Ionescu TI, Taverne RH, Houweling PL, Drost RH, Nuijten S, Van Rossum J. Pharmacokinetic study of extradural and intrathecal sufentanil anaesthesia for major surgery. Br J Anaesth. 1991;66: 458–464. 10.1093/bja/66.4.458 1827340

[24] Slankamenac K, Graf R, Barkun J, Puhan MA, Clavien P-A. The comprehensive complication index: a novel continuous scale to measure surgical morbidity. Ann Surg. 2013;258: 1–7. 10.1097/SLA.0b013e318296c732 23728278

[25] Slankamenac K, Nederlof N, Pessaux P, de Jonge J, Wijnhoven BPL, Breitenstein S, et al The comprehensive complication index: a novel and more sensitive endpoint for assessing outcome and reducing sample size in randomized controlled trials. Ann Surg. 2014;260: 757–762; discussion 762–763. 10.1097/SLA.0000000000000948 25379846

[26] Mangram AJ, Horan TC, Pearson ML, Silver LC, Jarvis WR. Guideline for Prevention of Surgical Site Infection, 1999. Centers for Disease Control and Prevention (CDC) Hospital Infection Control Practices Advisory Committee. Am J Infect Control. 1999;27: 97–132; quiz 133–134; discussion 96. 10196487

[27] Probst P, Haller S, Bruckner T, Ulrich A, Strobel O, Hackert T, et al Prospective trial to evaluate the prognostic value of different nutritional assessment scores in pancreatic surgery (NURIMAS Pancreas). Br J Surg. 2017;104: 1053–1062. 10.1002/bjs.10525 28369809

[28] Dexter F. Wilcoxon-Mann-Whitney test used for data that are not normally distributed. Anesth Analg. 2013;117: 537–538. 10.1213/ANE.0b013e31829ed28f 23966647

[29] Craig P, Dieppe P, Macintyre S, Michie S, Nazareth I, Petticrew M. Developing and evaluating complex interventions: the new Medical Research Council guidance. Int J Nurs Stud. 2013;50: 587–592. 10.1016/j.ijnurstu.2012.09.010 23159157

[30] Bell R, Ward D, Jeffery J, Toogood GJ, Lodge JpA, Rao K, et al A Randomized Controlled Trial Comparing Epidural Analgesia Versus Continuous Local Anesthetic Infiltration Via Abdominal Wound Catheter in Open Liver Resection. Ann Surg. 2018 10.1097/SLA.0000000000002988 30080727

[31] Mouawad NJ, Leichtle SW, Kaoutzanis C, Welch K, Winter S, Lampman R, et al Pain control with continuous infusion preperitoneal wound catheters versus continuous epidural analgesia in colon and rectal surgery: A randomized controlled trial. Am J Surg. 2018;215: 570–576. 10.1016/j.amjsurg.2017.06.031 28688514

[32] Aloia TA, Kim BJ, Segraves-Chun YS, Cata JP, Truty MJ, Shi Q, et al A Randomized Controlled Trial of Postoperative Thoracic Epidural Analgesia Versus Intravenous Patient Controlled Analgesia after Major Hepatopancreatobiliary Surgery. Ann Surg. 2017;266: 545–554. 10.1097/SLA.0000000000002386 28746153 PMC5784834

[33] Liu SS, Wu CL. The effect of analgesic technique on postoperative patient-reported outcomes including analgesia: a systematic review. Anesth Analg. 2007;105: 789–808. 10.1213/01.ane.0000278089.16848.1e 17717242

[34] Werawatganon T, Charuluxanun S. Patient controlled intravenous opioid analgesia versus continuous epidural analgesia for pain after intra-abdominal surgery. Cochrane Database Syst Rev. 2005; CD004088 10.1002/14651858.CD004088.pub2 15674928

[35] Bartley EJ, Fillingim RB. Sex differences in pain: a brief review of clinical and experimental findings. Br J Anaesth. 2013;111: 52–58. 10.1093/bja/aet127 23794645 PMC3690315

[36] Slankamenac K, Slankamenac M, Schlegel A, Nocito A, Rickenbacher A, Clavien P-A, et al Impact of postoperative complications on readmission and long-term survival in patients following surgery for colorectal cancer. Int J Colorectal Dis. 2017;32: 805–811. 10.1007/s00384-017-2811-y 28411352

[37] Ballantyne JC, Carr DB, deFerranti S, Suarez T, Lau J, Chalmers TC, et al The comparative effects of postoperative analgesic therapies on pulmonary outcome: cumulative meta-analyses of randomized, controlled trials. Anesth Analg. 1998;86: 598–612. 10.1097/00000539-199803000-00032 9495424

[38] AWMF. S3-Leitlinie Prophylaxe der venösen Thromboembolie (VTE). AWMF-Leitlinien-Register Nr. 003/001; 2009.

[39] Probst P, Grummich K, Heger P, Zaschke S, Knebel P, Ulrich A, et al Blinding in randomized controlled trials in general and abdominal surgery: protocol for a systematic review and empirical study. Syst Rev. 2016;5: 48 10.1186/s13643-016-0226-4 27012940 PMC4806514

[40] Cook TM, Counsell D, Wildsmith J a. W, Royal College of Anaesthetists Third National Audit Project. Major complications of central neuraxial block: report on the Third National Audit Project of the Royal College of Anaesthetists. Br J Anaesth. 2009;102: 179–190. 10.1093/bja/aen360 19139027

