# Supplementary figures and images for: Continuous wound infiltration versus epidural analgesia for midline abdominal incisions – a randomized-controlled pilot trial (Painless-Pilot trial; DRKS Number: DRKS00008023)
Source: PLoS One. 2020 Mar 6;15(3):e0229898. doi: 10.1371/journal.pone.0229898 (PMC7059935; doi:10.1371/journal.pone.0229898)

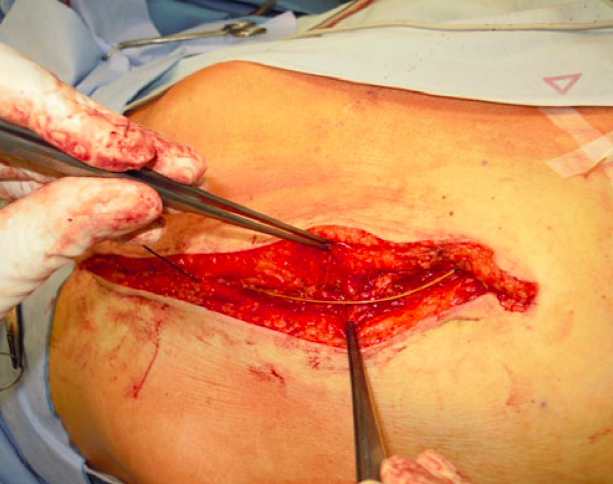

Supplement: S1 Fig — Reprinted with permission from Mann V. et al. Chirurg. 2011 Oct;82(10):906–12. (TIF) [file pone.0229898.s002.tif]

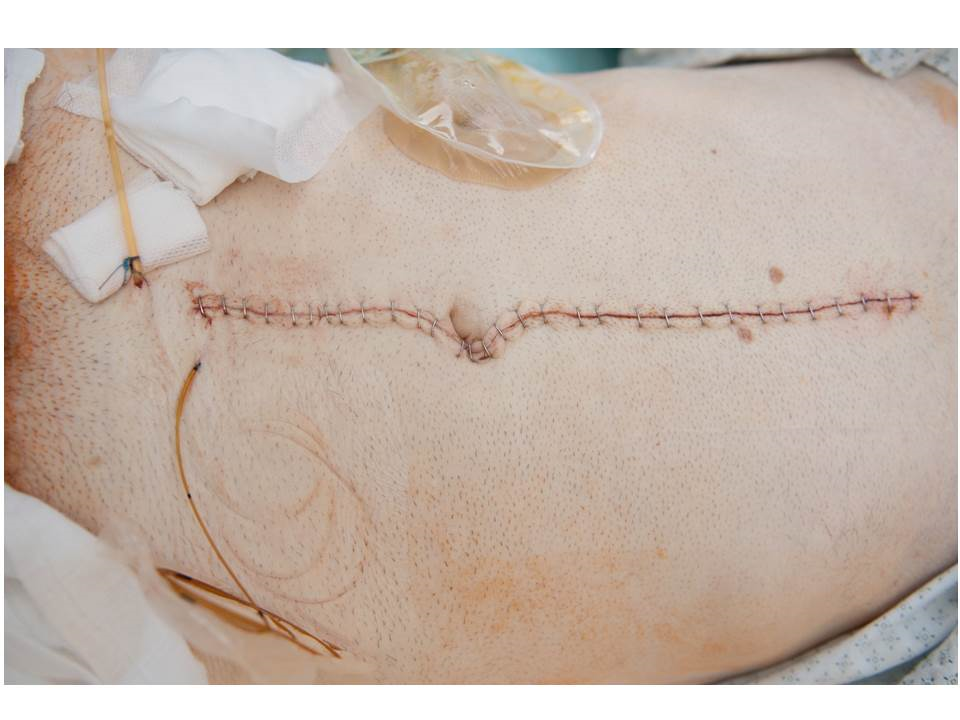

Supplement: S2 Fig — (TIF) [file pone.0229898.s003.tif]
