# Supplementary material for: Continuous wound infiltration versus epidural analgesia for midline abdominal incisions – a randomized-controlled pilot trial (Painless-Pilot trial; DRKS Number: DRKS00008023)
Source: PLoS One. 2020 Mar 6;15(3):e0229898. doi: 10.1371/journal.pone.0229898 (PMC7059935; doi:10.1371/journal.pone.0229898)
Supplement: S2 Table — (DOCX) [file pone.0229898.s005.docx]

**Supplement 5**

|  | CWI | EPA |
| --- | --- | --- |
| L-incision | 2 | 0 |
| Laparoscopy | 3 | 1 |
| No indication due to short operation time | 0 | 1 |
| Withdraw of IC | 0 | 2 |

**S5 table.** Reasons for exclusion from ITT set per treatment group. CWI: continuous wound infiltration. EPA: epidural analgesia. IC: informed consent.
