# Supplementary material for: Continuous wound infiltration versus epidural analgesia for midline abdominal incisions – a randomized-controlled pilot trial (Painless-Pilot trial; DRKS Number: DRKS00008023)
Source: PLoS One. 2020 Mar 6;15(3):e0229898. doi: 10.1371/journal.pone.0229898 (PMC7059935; doi:10.1371/journal.pone.0229898)
Supplement: S1 Protocol — (DOC) [file pone.0229898.s006.doc]

| **Clinical Trial Protocol** |
| --- |
| **Randomized-controlled pilot trial to compare continuous wound infiltration with local anesthetics via elastomer pump versus epidural analgesia following elective major abdominal surgery via midline laparotomy – PAINLESS-Pilot** |
| trial registration number |
| *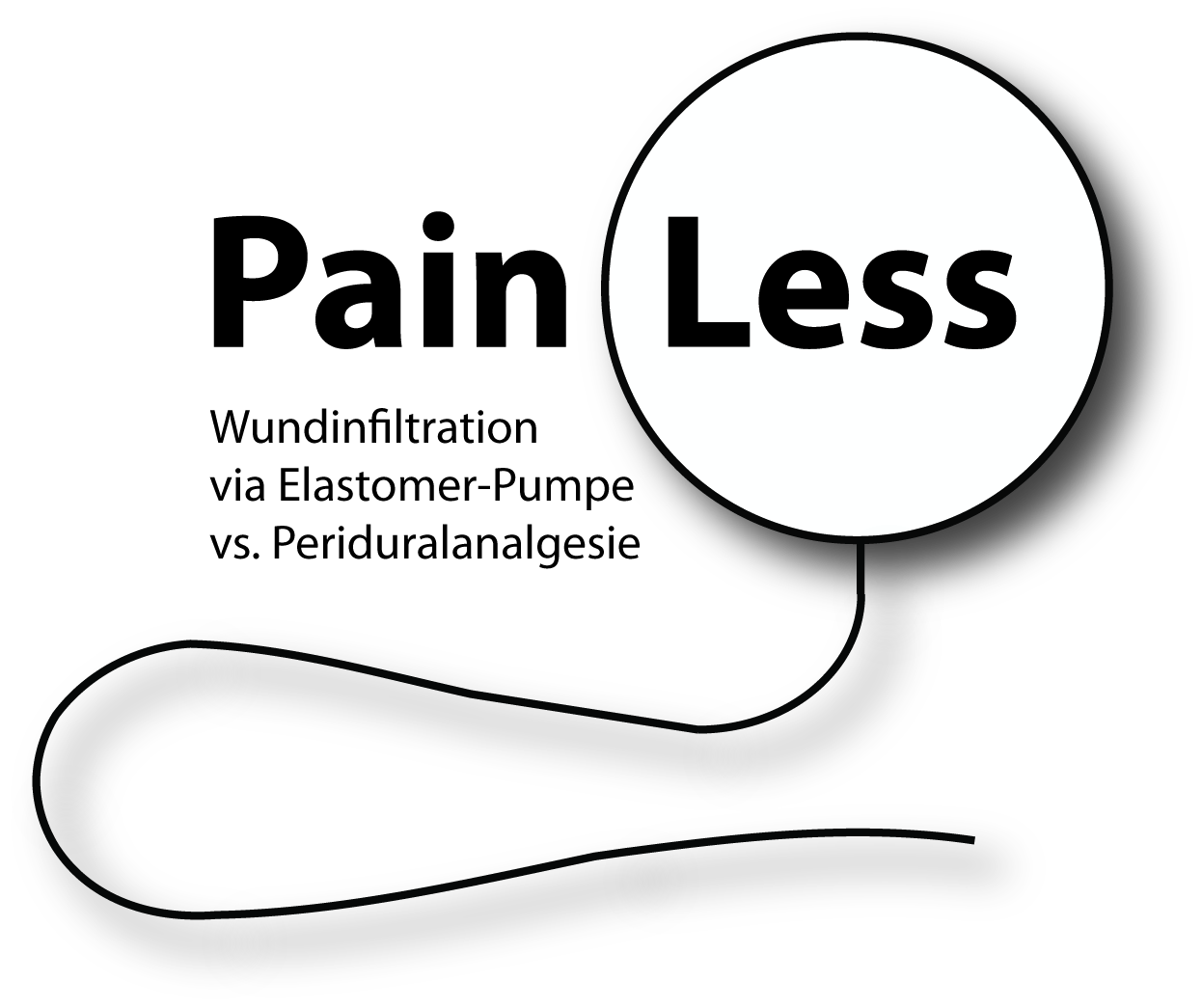*  The information in this trial protocol is strictly confidential. It is for the use of the sponsor, investigator, trial personnel, ethics committee, the authorities, and trial subjects only. This trial protocol may not be passed on to third parties without the agreement of the Interdisciplinary Study Centre Anesthesiology/Surgery (ISA/C). |
| Funding:  *Heidelberger Stiftung Chirurgie 2015* |

**Roles and Responsibilities**

**Principal Investigator**

PD Dr. André L. Mihaljevic, M.Sc.

Department of General, Visceral and Transplantation Surgery

Heidelberg University Hospital

Im Neuenheimer Feld 110, 69120 Heidelberg, Germany

mihaljevic@uni-heidelberg.de

**Coordinating Investigator**

Rosa Klotz

Department of General, Visceral and Transplantation Surgery

Heidelberg University Hospital

Im Neuenheimer Feld 110, 69120 Heidelberg, Germany

[Rosa.Klotz@med.uni-heidelberg.de](mailto:Rosa.Klotz@med.uni-heidelberg.de)

**ISA/C**

Prof. Dr. Markus Weigand

Prof. Dr. Stefan Hofer

Prof. Dr. Johann Motsch

Interdisciplinary Study Centre of Anesthesiology/Surgery (ISA/C)

Department of Anesthesiology

Heidelberg University Hospital

Im Neuenheimer Feld 110, 69120 Heidelberg, Germany

[Markus.Weigand@med.uni-heidelberg.de](mailto:Markus.Weigand@med.uni-heidelberg.de)

Dr. Phillip Knebel

Interdisciplinary Study Centre of Anesthesiology/Surgery (ISA/C)

Department of General, Visceral and Transplantation Surgery

Heidelberg University Hospital

Im Neuenheimer Feld 110, 69120 Heidelberg, Germany

[Phillip.Knebel@med.uni-heidelberg.de](mailto:Phillip.Knebel@med.uni-heidelberg.de)

**SDGC**

PD Dr. Markus K. Diener

Study Centre of the German Surgical Society (SDGC)

Im Neuenheimer Feld 110, 69120 Heidelberg, Germany

Markus.Diener@med.uni-heidelberg.de

**Biometrician**

Dr. rer. nat. Thomas Bruckner

Institute of Medical Biometry and Informatics

Im Neuenheimer Feld 305, 69120 Heidelberg, Germany

[bruckner@imbi.uni-heidelberg.de](mailto:bruckner@imbi.uni-heidelberg.de)

**Writing committee**

Markus Diener, Stefan Hofer, Rosa Klotz, Phillip Knebel, Andre Mihaljevic, Johann Motsch, Birgit Trierweiler-Hauke, Markus Weigand

**Signatures**

|  |  |  |  |  |
| --- | --- | --- | --- | --- |
| Principal Investigator  *Department of general, visceral and transplantation surgery, Heidelberg university hospital* |  | Signature |  | Date |

|  |  |  |  |  |
| --- | --- | --- | --- | --- |
| Biostatistician  *Institute of Medical Biometry and Informatics, University of Heidelberg* |  | Signature |  | Date |

Table of Contents

[SYNOPSIS 7](#__RefHeading___Toc415042557)

[FLOW CHART 9](#__RefHeading___Toc415042558)

[Abbreviations 10](#__RefHeading___Toc415042559)

[1.1 Scientific background 11](#__RefHeading___Toc415042560)

[1.2 Trial rationale 11](#__RefHeading___Toc415042561)

[1.3 Objectives 12](#__RefHeading___Toc415042562)

[1.4 Trial design 12](#__RefHeading___Toc415042563)

[1.5 Trial duration and schedule 13](#__RefHeading___Toc415042564)

[2. Trial Conduct 13](#__RefHeading___Toc415042565)

[2.1 Eligibility criteria 13](#__RefHeading___Toc415042566)

[2.1.1 Number of patients and trial sites 14](#__RefHeading___Toc415042577)

[2.1.2 Criteria for withdrawal of patients 14](#__RefHeading___Toc415042579)

[2.1.3 Premature closure of the trial 14](#__RefHeading___Toc415042580)

[2.2 Intervention(s) 15](#__RefHeading___Toc415042581)

[2.2.1 Description of trial interventions 15](#__RefHeading___Toc415042582)

[2.2.2 Benefits and risks of trial interventions 16](#__RefHeading___Toc415042583)

[2.2.3 Assignment of intervention and randomization 16](#__RefHeading___Toc415042584)

[2.2.4 Blinding 16](#__RefHeading___Toc415042585)

[2.3. Outcomes/endpoints 17](#__RefHeading___Toc415042587)

[2.3.1. outcome and assessment of outcome 17](#__RefHeading___Toc415042588)

[2.3.2 Definition of complications 17](#__RefHeading___Toc415042589)

[2.4. Patient schedule and documentation 18](#__RefHeading___Toc415042590)

[2.4.1 Patient timeline 18](#__RefHeading___Toc415042591)

[2.4.2 Description of trial visits 19](#__RefHeading___Toc415042592)

[3. Data Management 21](#__RefHeading___Toc415042593)

[4. Statistical Procedures 22](#__RefHeading___Toc415042594)

[4.1 Sample size calculation 22](#__RefHeading___Toc415042595)

[4.2 Analysis variables and statistical methods 22](#__RefHeading___Toc415042596)

[5. Quality Assurance 23](#__RefHeading___Toc415042597)

[5.1 Clinical data monitoring 23](#__RefHeading___Toc415042598)

[5.2 Assessment of Safety 23](#__RefHeading___Toc415042599)

[5.2.1 Data safety and monitoring board 23](#__RefHeading___Toc415042600)

[5.2.3 Definition, documentation and classification of Serious Adverse Events (SAE) 23](#__RefHeading___Toc415042604)

[5.3 Direct access to source data/documents 26](#__RefHeading___Toc415042605)

[6. Ethical and Legal Aspects 26](#__RefHeading___Toc415042606)

[6.1 Declaration of Helsinki 26](#__RefHeading___Toc415042607)

[6.2 Good Clinical Practice 26](#__RefHeading___Toc415042609)

[6.3 Patient information and informed consent 26](#__RefHeading___Toc415042610)

[6.4 Confidentiality 27](#__RefHeading___Toc415042611)

[6.5 Responsibilities of investigator 27](#__RefHeading___Toc415042612)

[6.6 Approval of trial protocol and amendments 27](#__RefHeading___Toc415042614)

[6.7 Ongoing information for independent Ethics Committee (IEC) 27](#__RefHeading___Toc415042615)

[6.8 Steering Committee 27](#__RefHeading___Toc415042616)

[6.9 Insurance 27](#__RefHeading___Toc415042617)

[7. Agreements 28](#__RefHeading___Toc415042618)

[7.1 Financing of the trial 28](#__RefHeading___Toc415042619)

[8. References 28](#__RefHeading___Toc415042620)

[9. Declaration of Investigator 31](#__RefHeading___Toc415042621)

[Appendices 32](#__RefHeading___Toc415042622)

**SYNOPSIS**

| **Coordinating Investigator** | Rosa Klotz  Klinik für Allgemein-, Viszeral und Transplantationschirurgie  Universitätsklinikum Heidelberg  Im Neuenheimer Feld 110  69120 Heidelberg |
| --- | --- |
| **Acronym** | PAINLESS-PILOT |
| **Title** | Randomized-controlled pilot trial to compare continuous wound infiltration (CWI) with local anesthetics via elastomer pump versus epidural analgesia (EPA) following elective major abdominal surgery |
| **Indication** | Patients undergoing elective upper abdominal surgery via a midline-laparotomy |
| **Objectives** | The primary aim of this study is to test the feasibility of assessing the postoperative mobility of patients via a pedometer in terms of number of steps per day. |
| **Trial design** | Randomized, controlled, unblinded, single center pilot study with two parallel study groups. |
| **Interventions** | Experimental intervention**:**  Epifascial placement of a CWI catheter (On-Q® PainBuster®, Vertrieb B. Braun, Melsungen) with Ropivacain 0.2% isobar at 5-10ml/h for 3 days via an elastomeric pump  Control intervention:  Thoracic EPA (Perifix®Komlett, Fa. B.Braun Melsungen) at TH 7/8-10/11 according to internal standards with postoperative Ropivacain 0,2% at 6-10ml/h for 3 days  Both groups:  Standardized baseline analgesia with 4x1g Novalgin or 4x1g Paracetamol i.v. and on demand analgesia with Oxycodone 5mg i.v. up to every four hours  Duration of intervention per patient:  3 days  Follow-up per patient:  30 days |
| **Eligibility criteria** | Key inclusion criteria:   - Elective upper abdominal surgery via a midline-laparotomy - Indication for EPA - Age ≥ 18 years - Written informed consent - Ability of subject to understand the nature and consequences of the clinical trial   Key exclusion criteria:   - Chronic pain and/or regular consumption of opioid analgesics - Contraindication for thoracic EPA or epifascial CWI - Inability to walk unaided and without adjuvants - Planned stoma placement - Hypersensitivity or allergy to one or more of the analgesic medications used in the trial - Pregnancy or breast-feeding - Participation in another intervention-trial with interference of intervention and outcome of this study |
| **Outcome(s)** | 1. Feasibility of assessing post-operative mobility with an OMRON Walking style Pro 2.0 pedometer (OMRON Medizintechnik Handelsgesellschaft mbH Mannheim). At the end of day 3 distance and number of steps for all three days will be read out separately 2. Feasibility of recruitment in the planned time frame 3. Evaluation of the comprehensive complication index (CCI) by examining the overall postoperative morbidity of patients in the study 4. Pain scores at rest and on movement 48 and 72 hours post-operatively according to the numeric rating scale (NRS) 5. Total amount of opioids administered during 0–24, 24–48 and 48-72 hours post-operatively 6. Time to first flatus/bowel movement 7. Body weight 8. Rate of treatment failure, defined as the need for the use of an intravenous patient-controlled analgesia (PCA) system 9. Rate of catheter-related complication 10. Rate of surgical side infection according to the definition of the Centre for Disease Control and Prevention (CDC) 11. Days of catheter-therapy (CWI/EPA) 12. Length of hospital stay   Assessment of Safety:  Rate of serious adverse events (SAEs) in both groups. |
| **Sample size** | To be assessed for eligibility: n = 120  To be allocated to trial: n = 70  To be analyzed: n = 60 |
| **Participating center** | Department of Surgery and Anesthesia, Heidelberg University Hospital,  Im Neuenheimer Feld 110, 69120 Heidelberg, Germany |
| **Statistical analysis** | Descriptive evaluation of the outcomes defined above including appropriate statistical tests  t-test to compare continuous data, chi-square-test to analyze categorical data  Safety:  Exploratory analyses of frequencies of complications SAEs |
| **Trial duration** | Trial preparation: 1 months  Recruitment: 5 months  Follow-up per patient: 30 days  Last patient in – last patient out: 6 months  Data collection, analysis, report: 3 months  Total length of trial: 10 months |
| **Financing of the trial** | Heidelberger Stiftung Chirurgie 2015 |

# FLOW CHART

Patients undergoing elective major abdominal surgery via midline-laparotomy

**n = 120**

**Compliant patients fulfilling inclusion criteria with**

**informed consent n = 70**

**Randomization**

**Experimental intervention (CWI) n = 35**

**n = 35**

**day 1**

**n = 50 excluded**

**(not fulfilling inclusion criteria)**

**Visit 4**

**Visit 2**

**Visit 3**

**Visit 1**

**day 2**

**day 3**

**Visit 5**

**To be analyzed**

**n = 30**

**Control intervention (EPA)**

**n = 35**

**day 1**

**day 2**

**day 3**

**Expected**

**drop-out rate**

**in each group**

**n = 5**

**To be analyzed**

**n = 30**

**day 7**

**day 7**

**day 30-35**

**day 30-35**

**Visit 6**

**Visit 7**

# Abbreviations

| **AE**  **CCI** | Adverse Event  Comprehensive Complication Index |
| --- | --- |
| **CDC** | Centre for Disease Control and Prevention |
| **CRF** | Case Report Form |
| **CWI** | Continuous wound infiltration |
| **EPA** | Epidural Analgesia |
| **GCP** | Good Clinical Practice |
| **ICH** | International Conference on Harmonization of Technical Requirements for Registration of Pharmaceuticals for Human Use |
| **IEC** | Independent Ethics Committee |
| **ISA/C**  **ITT**  **NRS** | Interdisciplinary Study Centre Anesthesiology/Surgery  Intention-To-Treat  Numeric Rating Scale |
| **PCA** | Patient controlled analgesia |
| **SAE** | Serious Adverse Event |
| **SDGC** | Study Center of German Surgical Society |
|  |  |

1. **INTRODUCTION**
   1. **1.1 Scientific background**
   2. **1.2 Trial rationale**

Globally, a total number of 234 million surgical interventions are performed every year . In Germany, 2 400 000 surgeries on the digestive organs are performed annually . Sufficient analgesia is inevitable for the perioperative management after these interventions and it reduces postoperative complications and the development of chronic pain .

In major abdominal surgery, different analgesic techniques such as systemic intravenous patient-controlled opioid therapy or locoregional techniques, such as EPA, are established standard procedures for effective perioperative pain management . Currently, following the German S3 guidelines, EPA is the first line therapy for postoperative analgesia in major abdominal surgery. These recommendations are based on a number of studies comparing EPA to systemic opioid therapy and showing a non-significant reduction of pulmonary complications for the EPA group . Furthermore, this effect could only be shown in patients undergoing major vascular surgery and for high risk patients . Besides, postulated advantages of EPA are enhanced pain control, reduced consumption of anesthetics, reduction of the surgical stress response and early bowel recovery . However, the effect of EPA on clinically relevant outcomes such as length of hospital stay was not proven and more recent studies demonstrate no difference in terms of morbidity and mortality .

In addition, EPA is associated with a number of drawbacks: a.) EPA is associated with rare, but serious adverse events like bleeding, infection and paraplegia ; b.) anticoagulation and coagulation disorders are a contraindication for EPA; c.) degenerative spine diseases inhibit the placement of an epidural catheter; d.) comorbidities like aortic-/mitral-valve stenosis, hypovolemia, increased cerebral pressure, sepsis etc. are contraindications for EPA; e.) a significant number of patients shows catheter dislodgement and treatment failure following EPA (failure rate 13 - 47% in experienced hands ); f.) EPA placement, care and removal are associated with significant personal and material costs; g.) through vasodilatation EPA can provoke hemodynamic instability in the setting of major abdominal surgery with possible large volume shifts and significant blood losses which is a risk factor for postoperative complications . Vasopressors and large amounts of fluids then administered to stabilize hypotension may impair the healing of gastrointestinal anastomoses .

These drawbacks of EPA have led to the development of new analgesic techniques. For instance, CWI with local anesthetics has been used to treat postoperative pain . Its potential advantages over EPA are: a.) easy placement and removal similar to subcutaneous drains; b.) the potential increased mobility of patients as no urinary catheter or heavy pumps are required; c.) reduced costs; d.) less adverse events (AEs) as no placement in the epidural space is required.

A number of trials have shown its equivalent analgesic effect following thoracic surgery . Consequently, for thoracic surgery, the PROSPECT (“procedure-specific postoperative pain management”)-group, an interdisciplinary work group of surgeons and anesthesiologists, recommends CWI as equivalent to EPA . Furthermore, it has been studied in abdominal surgery in a number of smaller trials . In terms of postoperative pain control CWI has been shown to be equivalent to EPA following abdominal surgery in a recent systematic review . Until now, no study has compared the overall postoperative morbidity with CWI compared to EPA. Non-inferiority in terms of post-operative complications together with already shown sufficient pain control of the CWI could lead to a therapeutic alternative for patients undergoing elective major abdominal surgery. Besides, the severe adverse effects such as epidural hematoma and the time and effort necessary for introducing the EPA could be avoided.

For the patient, CWI is associated with less technical devices than EPA and does not require a urinary catheter. Thus, the CWI might provide a potential superiority regarding mobilization leading to earlier postoperative mobilization, reducing pulmonary and thrombotic complications and ultimately thereby enhancing recovery.

In this pilot trial we aim to assess the mobility of patients following CWI or EPA placement and major abdominal surgery. Mobility will be measured via a pedometer (OMRON Medizintechnik Handelsgesellschaft mbH Mannheim) which continuously records the number of steps taken and distance covered and has been employed in several trials before . As secondary endpoints we will analyze postoperative pain via the NRS. In order to assess postoperative complications the CCI will be calculated for each patient using the Clavien-Dindo classification of postoperative morbidity .

It is planned to use the results of this pilot trial as basis for a multicenter study to evaluate the efficacy of CWI in comparison to EPA.

- 1. **1.3 Objectives**

The PAINLESS-Pilot will assess the feasibility of evaluating postoperative mobility of patients via a pedometer following major abdominal surgery and postoperative pain control with CWI vs. EPA.

- 1. **1.4 Trial design**

PAINLESS-Pilot is a randomized, controlled, unblinded, single center pilot study with two parallel study groups.

- 1. **1.5 Trial duration and schedule**

Duration of the entire trial (10 months) is as follows:

1. Trial preparation: 1 month
2. Trial performance: First patient in to last patient out: 6 months
   - 1. Recruitment phase: 5 months
     2. Follow-up per patient: 30 days postoperatively
3. Database closure: 1 month
4. Statistical analysis: 1 month
5. Report: 1 month

The trial is closed with the last patient finishing the last follow-up visit.

Table 1: Trial time plan

| Inclusion of first patient | Mai 2015 |
| --- | --- |
| Inclusion of last patient | September 2015 |
| End of trial last patient | October 2015 |
| Database closure | November 2015 |
| Statistical analysis | December 2015 |
| Report | January 2016 |

1. **Trial Conduct**
   1. **2.1 Eligibility criteria**
   2. *Key inclusion criteria:*

- Elective upper abdominal surgery via midline-laparotomy
- Indication for EPA
- Age ≥ 18 years
- Written informed consent
- Ability to understand the character and individual consequences of the clinical trial
  1. *Key exclusion criteria:*
- Chronic pain and/or regular consumption of opioid analgesics
- Contraindication for EPA or CWI
- Inability to walk unaided and without adjuvants
- Planned stoma placement
- Hypersensitivity or allergy to one or more of the analgesic medications used in the trial
- Pregnancy or breast-feeding
- Participation in another intervention-trial with interference of intervention and outcome of this study
  - 1. **Number of patients and trial sites**

Since the aim of this single center study is to assess the feasibility of the pedometer, patient recruitment and the CCI no sample size calculation has been performed. 60 Patients (30 patients in each group) were deemed necessary to draw valid conclusions in terms of feasibility. Due to the very broad inclusion criteria, the limited number of exclusion criteria and the comprehensible nature of the trial, not more than 50 patients are expected to be screening failures resulting in a total number of patients that need to be screened for eligibility of 120 (see flow chart). After randomized a dropout rate of 10 patients (5 in each study arm) is expected.

- - 1. **Criteria for withdrawal of patients**

Patients are free to leave the trial at any time and without giving reasons for their decision. Subjects may be withdrawn from the trial for the following reasons: (a) At their own request or (b) If, in the investigator’s opinion, continuation of the trial would be detrimental to the subject’s well-being. In case of (b), the reason for withdrawal must be recorded in the case report form (CRF) and in the patient’s medical records.

All ongoing SAEs of withdrawn subjects have to be followed up until no more signs and symptoms are verifiable or the patient is in stable condition.

- - 1. **Premature closure of the trial**

The trial may be prematurely closed by the principal investigator in consultation with the responsible biometrician.

Reasons that may necessitate a termination of the trial include the following:

- The incidence or severity of SAEs/morbidity in this trial indicates a potential health hazard caused by the study treatment.
- It appears that patients’ enrolment is unsatisfactory with respect to quality and/or quantity or data recording is severely inaccurate and/or incomplete.
- External evidence demanding a termination of the trial.

The independent ethics committee (IEC) must then be informed.

- 1. **2.2 Intervention(s)**

**2.2.1 Description of trial interventions**

Patients in the intervention group will receive a CWI catheter which will be placed suprafascially in the surgical site of the midline-laparotomy at the end of the surgery after closure of the abdominal fascia and before the closure of the skin. The catheter must cover the complete length of the incision. If necessary, more than one catheter can be used. We will use a On-Q® PainBuster®, Vertrieb B.Braun, Melsungen) with Ropivacain 0.2% isobar at 5-10ml/h for 3 days which is connected to an elastomeric pump releasing the local anaesthetic continuously.


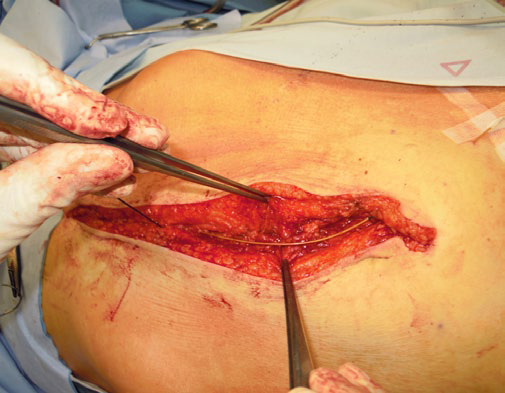


Figure 1. Suprafascial placement of CWI catheter

In the control group patients will preoperatively receive a thoracic EPA (Perifix®Komlett, Fa. B.Braun Melsungen) at TH 7/8–10/11 according to internal standards with Ropivacain 0.2% at 6-10ml/h administered postoperatively for 3 days.

In both groups patients will receive a standardized additional pain medication with 1g Metamizol or Paracetamol four times a day, and intravenous Oxycodone 5mg up to every 4 hours on demand. Both the CWI and the EPA should be removed at the third postoperative day. In case of treatment failure (defined as pain not controllable despite of intravenous Oxycodone) a systemic opioid PCA will be used. Other analgesic medication will not be given, unless the patient’s pain is not controllable with the recommended pain medication.

Abdominal wound closure will be performed in a standardized manner according as recommended by Israelsson . A running suture with Monomax® (Fa. B. Braun Melsungen) with a suture length to wound length ratio of at least 4 and practising a short stitch length (ie, placing stitches <10 mm from the wound edge) will be performed for fascial closure. No subcutaneous sutures will be placed and skin suture will be stapled.

Overall postoperative management will be performed identically in both intervention groups according to in-hospital standards.

**2.2.2 Benefits and risks of trial interventions**

Patients in both groups will receive a state-of-the-art postoperative pain-management. EPA is recommended in current national S3 guidelines in Germany . Patients in the intervention group will receive CWI, which has been shown to be as effective as EPA in terms of pain control . In thoracic surgery the CWI is already accepted as first line therapy and equivalent to thoracic EPA by an international interdisciplinary study group including surgeons and anesthesiologists .

Both analgesic methods used in this study have their specific risks and benefits. As mentioned above, on the one hand, EPA shows a weak, but not statistically significant tendency to reduce pulmonary complications in a subgroup analysis compared to systemic opioid therapy in patients undergoing major vascular surgery and in high risk patients . On the other hand, failure of the epidural anesthesia and analgesia is a frequent clinical problem (failure rate 13 - 47% in experienced hands) . Furthermore, the incidence of epidural hematoma (1:6,628) and paraplegia is rare but dangerous and occurs more frequently than previously thought.

For CWI compared to the EPA no difference in terms of overall morbidity could be shown. Solely, the incidence of urinary retention was significantly lower in the wound infiltration group than in the EPA group . Incidence of surgical site infections showed no significant difference between CWI and EPA , but it is known that local anesthetics show a bactericidal effect and lead to less inflammation .

**2.2.3 Assignment of intervention and randomization**

The randomization sequence will be computer-generated using the standard software. Randomization will be performed blockwise. The randomization number will be brought to the operation theatre in sealed opaque envelopes, where randomization (visit 2, see *flow-chart*) will be performed before anesthetization.

**2.2.4 Blinding**

Blinding patients and observers to the intervention will not be possible as the insertion of the epidural catheter is performed when the patient is awake. Moreover, postoperatively the difference between wound catheter and epidural catheter will be obvious to patient and observer. However, risk of bias due to non-blinding will be low as the pedometer gives an objective recording mobility. Furthermore, some of the other endpoints like complication frequency according to Clavien-Dindo are objective and do not require blinding.

- 1. **Outcomes/endpoints**
     1. **outcome and assessment of outcome**

1. Feasibility of assessing post-operative mobility with a pedometer. For this purpose patients have to wear the pedometer the complete day, which will be monitored during clinical visits on day 1, 2 and 3. An OMRON Walking style Pro 2.0 pedometer (OMRON Medizintechnik Handelsgesellschaft mbH Mannheim) will be used and placed on hip level of the patient using a kinesio taping. At the end of day 3 distance and number of steps for all three days will be read out separately.
2. Feasibility of recruitment in the planned time frame (in 5 months: n = 120 to be assessed for eligibility, n = 70 to be allocated to trial, n = 60 to be analyzed). This endpoint will be assessed at the end of the study.
3. Evaluation of the CCI by examining the overall postoperative morbidity of patients in the study. All complications will be assessed prospectively at each clinical visit and from the patients’ file. Severity of each complication will be classified according to the Clavien-Dindo classification as described under 2.3.2. Finally, the CCI will be calculated for each patient as has been described before (http://www.assessurgery.com/) .
4. Pain scores at rest and on movement 48 and 72 hours post-operatively according to the NRS.
5. Total amount of opioids administered during 0–24, 24–48 and 48-72 hours post-operatively (converted to equivalence dose of morphine).
6. Time to first flatus/bowel movement (counting in days from the first postoperative day).
7. Body weight (in kilogram)
8. Rate of treatment failure, defined as the need for the use of an intravenous PCA system.
9. Rate of catheter-related complication (e.g. neuroaxial hematoma (bleeding into the vertebral canal confirmed by MRI and/or CT scan), dislodgment, infection) will be recorded.
10. Rate of surgical side infection according to the definition of the CDC will be evaluated.
11. Days of catheter-therapy (CWI/PDA, counting in days from the first postoperative day).
12. Length of hospital stay.
13. Rate of SAEs in both groups.

**2.3.2 Definition of complications**

The following table shows a classification system to grade complications by severity based on the type and invasiveness of the treatment needed to treat a complication proposed by Clavien and Dindo et al. .

| **Grade** | **Definition** |
| --- | --- |
| Grade I | Any deviation from the normal postoperative course without the need for pharmacological treatment or surgical, endoscopic, and radiological interventions.  Allowed therapeutic regimens are: drugs as antiemetics, antipyretics, analgetics, diuretics and electrolytes and physiotherapy. This grade also includes wound infections opened at the bedside. |
| Grade II | Requiring pharmacological treatment with drugs other than such allowed for grade I complications.  Blood transfusions and total parenteral nutrition are also included. |
| Grade III  Grade IIIa  Grade IIIb | Requiring surgical, endoscopic or radiological intervention.  Intervention not under general anesthesia.  Intervention under general anesthesia. |
| Grade IV  Grade IVa  Grade IVb | Life-threatening complication (including CNS complications)* requiring IC/ICU-management  Single organ dysfunction (including dialysis)  Multi organ dysfunction |
| Grade V | Death of a patient |
| Suffix "d" | If the patient suffers from a complication at the time of discharge, the suffix "d" (for ‘disability’) is added to the respective grade of complication.  This label indicates the need for a follow-up to fully evaluate the complication. |

*Brain hemorrhage, ischemic stroke, subarrachnoidal bleeding, but excluding transient ischemic attacks

**Table 1.** Clavien-Dindo Classification of surgical complications

The CCI is calculated as the sum of all complications that are weighted for their severity (multiplication of the median reference values from patients and physicians). The final formula yields a continuous scale to rank the severity of any combination of complications from 0 to 100 in a single patient. The CCI can be readily computed on the basis of tabulated complications according to the Clavien-Dindo classification (available at [www.assessurgery.com](http://www.assessurgery.com/)) .

- 1. **Patient schedule and documentation**

**2.4.1 Patient timeline**

Patients scheduled for elective major abdominal surgery via a midline-laparotomy are screened four weeks before operation to one day before operation (visit 1). Patients fulfilling inclusion/exclusion criteria who consent to take part in the trial, demographic data and medical history are assessed and documented (visit 1). Patients are randomized in the operation theater before anaesthesia (visit 2; randomization, surgery). Intraoperative parameters are assessed during surgery (visit 2). Patients are planned to follow-up at postoperative day 1, 2 and 3 (visit 3-5) for evaluation of primary and secondary endpoints as well as SAEs and postoperative day 7, and 30-35 days (visit 6-7) for evaluation of secondary endpoints and SAEs*.*

Duration of trial intervention per patient: 3 days

Duration of trial per patient: 30 days (+5 days)

**2.4.2 Description of trial visits**

**Visit 1 (screening, informed consent)**

Patients are screened preoperatively. All consecutive patients are enlisted in a screening list. Reasons for non-enrollment must be stated. Patients are enrolled given their ability to understand the extent and nature of the PAINLESS-Pilot trial as well as their written informed consent after detailed patient information. The following data items are collected in visit 1:

1. Documentation of all consecutive patients in a screening list
2. Check inclusion/exclusion criteria
3. Written informed consent
4. Demographic data and medical history:
5. Date
6. Age
7. Size and weight to calculate BMI
8. Sex
9. Current pain medication
10. Underlying disease

**Visit 2 (randomization, anaesthetization and surgery, day 0)**

Surgery is performed according to local standard. Randomization will be performed before anesthetization as described in section 2.2.3. Abdominal wall closure will subsequently be performed as described in section 2.2.1.

The following data items need to be documented:

1. Date
2. Type of surgery
3. Drain insertion (number and type)
4. Length of midline-laparotomy (in cm)
5. Type of abdominal fascial closure
6. Duration of operation (in min, according to documentation, incision-to-skin closure)
7. Duration of induction of anesthesia (in min, according to anesthesia protocol, first contact anesthesiologist-patient to information of surgeons “patient ready for surgery”)
8. Intraoperative SAE documentation (according to 5.2.3)
9. Attachment of pedometer to the patient´s hip.

**Visit 3 (postoperative day 1)**

Visit 3-5 have equivalent contents, but are performed at different postoperative days. They are performed to document number of steps, perioperative complications and morbidity. The following data items need to be documented:

1. Number of steps and distance covered (in meter) over 24 hours (0.00am-24.00pm) assessed by pedometer
2. Postoperative complications according to Clavien-Dindo . Documentation sheet see section 2.3.2
3. Pain scores at rest and with movement according to the NRS
4. Total amount of opioids administered during 24 hours
5. Time to first flatus and time to first bowel movement (in days)
6. Body weight (in kilogram)
7. Rate of treatment failure, defined as indication for the use of a PCA system
8. Rate of catheter-related complication
9. Rate of surgical site infection according to the definition of CDC
10. Days of catheter-therapy (CWI/PDA, counting in days from the first postoperative day)
11. Rate of SAEs (according to 5.2.3)

**Visit 4 (postoperative day 2)**

Visit 4 has the same content as visit 3 (see above), but has to be performed at postoperative day 2.

**Visit 5 (postoperative day 3)**

Visit 5 has the same content as visit 3 (see above), but has to be performed at postoperative day 3.

**Visit 6 (postoperative day 7)**

Visit 6-7 have equivalent contents, but are performed at different postoperative days. The following data items need to be documented:

1. Postoperative complications according to Clavien-Dindo
2. Time to first flatus and to first bowel movement (in days)
3. Body weight (in kilogram)
4. Rate of treatment failure, defined as indication for the use of a PCA system
5. Rate of catheter-related complications
6. Rate of surgical side infection according to the definition of the CDC
7. Days of catheter-therapy (CWI/PDA, counting in days from the first postoperative day)
8. Length of hospital stay (counting in days from the first postoperative day, objectively)
9. Rate of AEs and SAEs in both groups according to appendix 3

**Visit 7 (postoperative day 30-35)**

Visit 7 has the same content as visit 6 (see above), but has to be performed 30-35 days after trial intervention.

The following table gives an overview about trial visits.

**Table 2: Trial visits and documented parameters**

| **Visit** | **1** | **2** | **3-5** | **6-7** |
| --- | --- | --- | --- | --- |
|  | Screening/  Consent | Randomization/  Intervention | Post-op day 1,2,3 | Post-op day 7 and 30-35 |
| Inclusion/exclusion criteria | **X** |  |  |  |
| Informed consent | **X** |  |  |  |
| Demographic data/ Medical history | **X** |  |  |  |
| Randomization |  | **X** |  |  |
| Surgery |  | **X** |  |  |
| Intraoperative parameters |  | **X** |  |  |
| Number of steps/distance covered |  |  | **X** |  |
| Pain scores (NRS) |  |  | **X** |  |
| Additional opioids |  |  | **X** |  |
| Time to first flatus/bowel movement |  |  | **X** | **X** |
| Body weight |  |  | **X** | **X** |
| Postoperative complications |  |  | **X** | **X** |
| Length of hospital stay |  |  |  | **X** |
| SAEs |  |  | **X** | **X** |

**3. Data Management**

All protocol-required information collected during the trial must be entered by the investigator, or designated representative, in the CRF. The investigator, or designated representative, should complete the CRF pages as soon as possible after information is collected, preferably on the same day that a trial subject is seen for an examination, treatment, or any other trial procedure. Any outstanding entries must be completed immediately after the final examination. An explanation should be given for all missing data.

The completed CRF must be reviewed and signed by the investigator named in the trial protocol or by a designated sub-investigator.

**4. Statistical Procedures**

**4.1 Sample size calculation**

Due to the broad inclusion criteria, the limited number of exclusion criteria and the comprehensible nature of the trial, no more than 50 patients are expected to be screened and not being included resulting in a total number of patients that need to be screened for eligibility of 120 (see flow chart). Then, 70 patients will be allocated to the trial and with a low expected drop-out number 60 patients are planned to be analyzed. Since this trial is a pilot study a sample size calculation with power and significance was not performed.

**4.2 Analysis variables and statistical methods**

*Analysis populations:*

*Intention to treat population (ITT):* The ITT population contains all patients that are randomized during visit 2 (randomization, intervention) independent of the intervention they receive (control or intervention). Analysis of all endpoints will occur as randomized, i.e. in the ITT population. Patients will be excluded from the ITT population if they do not fulfill preoperative or intraoperative inclusion criteria and were thus randomized by mistake (e.g. patients with laparoscopic interventions or transverse laparotomy).

No replacement or imputation of missing data will be done.

*Complete case population (CC):* The complete case population consists of all patients of the ITT population that have undergone visit 7 (30 days follow-up, analysis as randomized).

*Per-protocol population (PP)*: The PP population contains all patients included in the ITT population as treated (not as randomized) and have no major protocol deviations.

*Safety Population:* The safety population contains all patients that have started visit 2 independent of the treatment they receive.

Detailed descriptive statistics will be used to describe the sample, including mean, standard deviation, minimum, median and maximum in case of continuous data, with absolute and relative frequencies in case of categorical data. Possible differences between groups in the formulated endpoints will be evaluated with appropriate statistical tests (t-test to compare continuous data, chi-square test with categorical data). No formal hypothesis testing will be done; all p-values are used as decretive statistics. Statistical graphics will be used for illustration of the findings.

**5. Quality Assurance**

**5.1 Clinical data monitoring**

During the clinical trial, quality control and quality assurance will be ensured via monitoring. All investigators agree that the monitor can visit the center before, during and after completion of the study to ensure that the study is conducted, recorded and reported according to the study protocol, relevant standard operating procedures, requirements of good clinical practice (GCP) and the applicable regulatory requirements. The aim of monitoring is to assure that all results and conclusions written in the final report can be drawn from the source data. This includes controlling of data filing and organisation of the study center as well as controlling of third parties and original documents.

**5.2 Assessment of Safety**

Safety analysis include frequency and severity of SAEs.

- - 1. **Data safety and monitoring board**

No data and safety monitoring board will be initiated due to expected short time course of the study.

- - 1. **Definition and documentation of Adverse Events (AE)**

An AE is defined as any untoward medical occurrence or experience in a patient that occurs following surgery and that can be or not related to the treatment. This can include any unfavorable and unintended signs, or symptoms, an abnormal laboratory finding (including blood tests, x-rays or scans) or a disease temporarily associated with the use of the protocol treatment (International Conference on Harmonization of Technical Requirements for Registration of Pharmaceuticals for Human Use (ICH) -GCP).

Documentation:

As PAINLESS-Pilot is a non-AMG, non-MPG trial AEs will not be recorded. Instead, during the postoperative period (visit 3-7) all postoperative complications will be documented according to Clavien-Dindo.

- - 1. **Definition, documentation and classification of Serious Adverse Events (SAE)**

A SAE is any AE occurring at any time during the period of observation, that

- results in death.
- is immediately life-threatening.
- requires or prolongs hospitalization.
- results in persistent or significant disability or incapacity.

*Classification of intensity*

- Mild: awareness of signs or symptoms, but easily tolerated and are of minor irritant type causing no loss of time from normal activities. Symptoms do not require therapy or a medical evaluation; signs and symptoms are transient.
- Moderate: Events introduce a low level of inconvenience or concern to the participant and may interfere with daily activities, but are usually improved by simple therapeutic measures; moderate experiences may cause some interference with functioning.
- Severe: Events interrupt the participant’s normal daily activities and generally require systemic drug therapy or other treatment; they are usually incapacitating.

*Classification of outcome*

The outcome of an SAE at the time of last contact with the subject is classified.

- On-going: Signs and symptoms of the SAE still exist at end of trial participation for the individual patient.
- Recovered completely: All signs and symptoms of SAE have disappeared.
- Recovered with sequelae: Acute signs and symptoms of SAE have disappeared; sequelae caused by the SAE still exist.
- Death: The SAE has caused the death of the patient. If a subject has suffered from more than one SAE, only the outcome for the SAE directly responsible for death is classified as ‘death‘, the other SAE are classified according to their specific outcome.
- Unknown: The outcome is not known or is implausible and there is no possibility to complete or verify the information.

*Classification of causality*

- Unrelated: An SAE that does not follow a reasonable temporal sequence from trial treatment and that is likely to have been produced by the subject’s clinical state
- Possibly related: An SAE that has a reasonable possibility that the event may have been caused by trial treatment. The SAE has a timely relationship to the trial treatment(s); however, follows no known pattern of response, and an alternative cause seems more likely or there is significant uncertainty about the cause of the event.
- Definitely related: If there is a reasonable probability that the event may have been caused by the trial intervention. A certain event has a strong temporal relationship and an alternative cause is unlikely.
- Not assessable: There is insufficient or incomplete evidence to make a clinical judgment of the causal relationship to the trial treatment.

*Classification of countermeasures*

Actions taken have to be documented on the SAE form.

All ongoing SAEs (also SAEs of withdrawn subjects) have to be followed up until no more signs and symptoms are verifiable or the patient is in a stable condition.

*Reporting of SAEs*

From the day the patient has been randomized until the regular end of trial at postoperative day 30 or until premature withdrawal of the patient, all SAEs must be documented on a “serious adverse event form” available in the investigator site file.

The SAE form contains: identification of the trial subject, attending physician and center, description of the SAE (event, beginning, intensity, duration, severity, outcome, causality to the intervention of the trial, treatment/interventions taken), date, and signature of the attending physician.

SAEs have to be reported to the coordinating investigator within 5 days after they have become known:

Rosa Klotz,

PD Dr. med. André L. Mihaljevic,

Department of General, Visceral and Transplantation Surgery

University Hospital Heidelberg

Im Neuenheimer Feld 110, 69120 Heidelberg

Fax: +49-(0)-6221-56-34794

The initial report must be as complete as possible including details of the current illness and SAE and an assessment of the causal relationship between the event and the trial treatment.

1. The original SAE report remains in the investigator site file at the participating center until final control by the monitor.
2. All incoming SAEs are checked by Study Center of the German Surgical Society (SDGC) safety regarding completeness, correctness, and plausibility. Queries are clarified directly with the investigator in the concerned center.
3. All SAEs are listed. For monitoring purposes, all necessary information on SAE reports is made available to the monitors.

The following SAEs **need not** be reported:

- SAEs occurring after giving informed consent but before operation
- Planned elective admission to hospital
- Planned elective surgery

**5.3 Direct access to source data/documents**

The participating clinical trial center permits trial-related monitoring audits and regulatory inspection(s), providing direct access to all necessary source data/documents.

**6. Ethical and Legal Aspects**

**6.1 Declaration of Helsinki**

The trial will be conducted according to the ethical principals layed out in the declaration of Helsinki (in its latest amendment Fortaleza, Brazil, October 2013) and the laws and regulations of the country (e.g. code of medical ethics [BOÄ]).

**6.2 Good Clinical Practice**

This trial protocol has been written and the trial will be conducted and analyzed in accordance with ICH E6 GCP to all relevant national and international rules and regulations.

**6.3 Patient information and informed consent**

All patients will be informed by an authorized investigator about the aims of the study, the possible risks, the procedures and possible hazards to which he/she will be exposed, and the machanism of treatment allocation. It will be emphasized that the patient is allowed to refuse further participation in the trial whenever he/she wants to.

This will not prejudice the patient’s subsequent care. Documented informed consent must be obtained for all patients included in the study before they are randomised in the study. The written informed consent form will be signed and personally dated by the patient according to the ICH guidelines on GCP. All patients will get sufficient time to decide upon the participation in this trial.

Furthermore, it is the responibility of the investigator to explain patients their duties within the trial.

**6.4 Confidentiality**

Patient data in the CRF will be pseudonymized. Patients will be informed as to the strict confidentiality of their data. However, medical records may be reviewed for trial purposes by authorized individuals other than their treating physician.

**6.5 Responsibilities of investigator**

The investigator should ensure that all persons assisting in the trial are adequately informed about the protocol, any amendments to the protocol, the trial treatments, and their trial-related duties and functions.

The investigator should maintain a list of sub-investigators and other appropriately qualified persons to whom he or she has delegated significant trial-related duties.

**6.6 Approval of trial protocol and amendments**

The trial protocol as well as the amendments have to be reviewed and signed by all parties.

Any changes of the protocol have to be disscussed and approved by all parties.

**6.7 Ongoing information for independent Ethics Committee (IEC)**

Once the trial has started, any changes have to be documented in a written amendment. They should be restricted to exceptional cases. Amendments then become part of the clinical trial protocol. The IEC must be informed of all subsequent protocol amendments. They must be evaluated to determine whether formal approval must be sought and whether the informed consent document should also be revised.

Since all anaesthesiological procedures examined in this trial are well established and in current daily use, no increased medical risks are expected for the participating patients. The IEC must be informed of the end of the trial.

**6.8 Steering Committee**

A steering committee will not be established due to the pilot nature of the trial.

**6.9 Insurance**

PAINLESS -Pilot trial is conducted according to the Medical Association's professional code (Musterberufsordnung der Bundesärztekammer) § 15. Therefore, there is no obligation for specific trial liability insurance in Germany.

**7. Agreements**

**7.1 Financing of the trial**

The trial will be financed by the “Stiftung Chirurgie Heidelberg”.

**8. References**

1. Mangram AJ, Horan TC, Pearson ML, Silver LC, Jarvis WR. Guideline for Prevention of Surgical Site Infection, 1999. American Journal of Infection Control. 1999;27(2):97-134.

2. Weiser TG, Makary MA, Haynes AB, Dziekan G, Berry WR, Gawande AA. Standardised metrics for global surgical surveillance. The Lancet. 2009;374(9695):1113-7.

3. Krankenhausstatistik F. Operationen und Prozeduren der vollstationären Patientinnen und Patienten in Krankenhäusern-Ausführliche Darstellung. Wiesbaden: Statistisches Bundesamt. 2009.

4. Kehlet H, Wilmore DW. Multimodal strategies to improve surgical outcome. The American journal of surgery. 2002;183(6):630-41.

5. Perkins FM, Kehlet H. Chronic pain as an outcome of surgery: a review of predictive factors. Anesthesiology. 2000;93(4):1123-33.

6. Wu CL, Cohen SR, Richman JM, Rowlingson AJ, Courpas GE, Cheung K, et al. Efficacy of postoperative patient-controlled and continuous infusion epidural analgesia versus intravenous patient-controlled analgesia with opioids: a meta-analysis. Anesthesiology. 2005;103(5):1079-88; quiz 109-10.

7. Ballantyne JC, Carr DB, deFerranti S, Suarez T, Lau J, Chalmers TC, et al. The comparative effects of postoperative analgesic therapies on pulmonary outcome: cumulative meta-analyses of randomized, controlled trials. Anesth Analg. 1998;86(3):598-612.

8. Liu SS, Wu CL. Effect of Postoperative Analgesia on Major Postoperative Complications: A Systematic Update of the Evidence. Anesthesia & Analgesia. 2007;104(3):689-702.

9. Panousis P, Heller AR, Koch T, Litz RJ. Epidural ropivacaine concentrations for intraoperative analgesia during major upper abdominal surgery: a prospective, randomized, double-blinded, placebo-controlled study. Anesthesia & analgesia. 2009;108(6):1971-6.

10. Gendall K, Kennedy R, Watson A, Frizelle F. The effect of epidural analgesia on postoperative outcome after colorectal surgery. Colorectal Disease. 2007;9(7):584-98.

11. Kooij FO, Schlack WS, Preckel B, Hollmann MW. Does Regional Analgesia for Major Surgery Improve Outcome? Focus on Epidural Analgesia. Anesthesia & Analgesia. 2014;119(3):740-4.

12. Revie EJ, McKeown DW, Wilson JA, Garden OJ, Wigmore SJ. Randomized clinical trial of local infiltration plus patient-controlled opiate analgesia vs. epidural analgesia following liver resection surgery. HPB. 2012;14(9):611-8.

13. Pöpping DM, Wenk M, Van Aken HK. [Neurologic complications after epidural analgesia]. Anasthesiologie, Intensivmedizin, Notfallmedizin, Schmerztherapie: AINS. 2012;47(5):336-43; quiz 44.

14. Hermanides J, Hollmann M, Stevens M, Lirk P. Failed epidural: causes and management. British journal of anaesthesia. 2012;109(2):144-54.

15. Gauss PDA, Jahn S, Eberhart L, Stahl W, Rockemann M, Georgieff M, et al. Kardioprotektion durch thorakale Periduralanästhesie? Der Anaesthesist. 2011;60(10):950-62.

16. Tassoudis V, Vretzakis G, Petsiti A, Stamatiou G, Bouzia K, Melekos M, et al. Impact of intraoperative hypotension on hospital stay in major abdominal surgery. Journal of anesthesia. 2011;25(4):492-9.

17. Rahbari N, Zimmermann J, Schmidt T, Koch M, Weigand M, Weitz J. Meta‐analysis of standard, restrictive and supplemental fluid administration in colorectal surgery. British Journal of Surgery. 2009;96(4):331-41.

18. Ventham N, Hughes M, O'Neill S, Johns N, Brady R, Wigmore S. Systematic review and meta‐analysis of continuous local anaesthetic wound infiltration versus epidural analgesia for postoperative pain following abdominal surgery. British Journal of Surgery. 2013;100(10):1280-9.

19. Kaiser AM, Zollinger A, De Lorenzi D, Largiadèr F, Weder W. Prospective, randomized comparison of extrapleural versus epidural analgesia for postthoracotomy pain. The Annals of Thoracic Surgery. 1998;66(2):367-72.

20. Davies RG, Myles PS, Graham JM. A comparison of the analgesic efficacy and side-effects of paravertebral vs epidural blockade for thoracotomy—a systematic review and meta-analysis of randomized trials. British Journal of Anaesthesia. 2006;96(4):418-26.

21. Joshi GP, Bonnet F, Shah R, Wilkinson RC, Camu F, Fischer B, et al. A Systematic Review of Randomized Trials Evaluating Regional Techniques for Postthoracotomy Analgesia. Anesthesia & Analgesia. 2008;107(3):1026-40.

22. Bertoglio S, Fabiani F, De Negri P, Corcione A, Merlo DF, Cafiero F, et al. The postoperative analgesic efficacy of preperitoneal continuous wound infusion compared to epidural continuous infusion with local anesthetics after colorectal cancer surgery: a randomized controlled multicenter study. Anesthesia & Analgesia. 2012;115(6):1442-50.

23. Niraj G, Kelkar A, Jeyapalan I, Graff‐Baker P, Williams O, Darbar A, et al. Comparison of analgesic efficacy of subcostal transversus abdominis plane blocks with epidural analgesia following upper abdominal surgery. Anaesthesia. 2011;66(6):465-71.

24. Almeida MCSd, Locks GdF, Gomes HP, Brunharo GM, Kauling A. Analgesia pós-operatória: comparação entre infusão continua de anestésico local e opioide via cateter peridural e infusão contínua de anestésico local via cateter na ferida operatória. Rev bras anestesiol. 2011;61:3.

25. Kambas A, Venetsanou F, Avloniti A, Giannakidou DM, Gourgoulis V, Draganidis D, et al. Pedometer determined physical activity and obesity prevalence of Greek children aged 4-6 years. Annals of human biology. 2014(0):1-6.

26. Giannakidou DM, Kambas A, Ageloussis N, Fatouros I, Christoforidis C, Venetsanou F, et al. The validity of two Omron pedometers during treadmill walking is speed dependent. Eur J Appl Physiol. 2012;112(1):49-57.

27. Hasson R, Haller J, Pober D, Staudenmayer J, Freedson P. Validity of the Omron HJ-112 pedometer during treadmill walking. Medicine+ Science in Sports+ Exercise. 2009;41(4):805.

28. Slankamenac K, Graf R, Barkun J, Puhan MA, Clavien P-A. The comprehensive complication index: a novel continuous scale to measure surgical morbidity. Annals of surgery. 2013;258(1):1-7.

29. Mann V, Mann S, Hecker A, Röhrig R, Müller M, Schwandner T, et al. Kontinuierliche lokale Wundinfusion mit Lokalanästhetika. Der Chirurg. 2011;82(10):906-12.

30. Millbourn D, Cengiz Y, Israelsson LA. Effect of stitch length on wound complications after closure of midline incisions: A randomized controlled trial. Archives of Surgery. 2009;144(11):1056-9.

31. für Schmerztherapie DIV. S3-Leitlinie ‚Behandlung akuter perioperativer und posttraumatischer Schmerzen—. Deutscher Ärzte-Verlag, Köln. 2008.

32. Hermanides J, Hollmann MW, Stevens MF, Lirk P. Failed epidural: causes and management. British Journal of Anaesthesia. 2012;109(2):144-54.

33. Volk T, Wolf A, Van Aken H, Burkle H, Wiebalck A, Steinfeldt T. Incidence of spinal haematoma after epidural puncture: analysis from the German network for safety in regional anaesthesia. Eur J Anaesthesiol. 2012;29(4):170-6.

34. Hahnenkamp K, Theilmeier G, Van Aken HK, Hoenemann CW. The Effects of Local Anesthetics on Perioperative Coagulation, Inflammation, and Microcirculation. Anesthesia & Analgesia. 2002;94(6).

35. Hollmann MW, Durieux ME. Local anesthetics and the inflammatory response: a new therapeutic indication? Anesthesiology. 2000;93(3):858-75.

36. Clavien PA, Sanabria JR, Strasberg SM. Proposed classification of complications of surgery with examples of utility in cholecystectomy. Surgery. 1992;111(5):518-26.

**9. Declaration of Investigator**

I have read the above trial protocol and I confirm that it contains all information to accordingly conduct the clinical trial. I pledge to conduct the clinical trial according to the protocol.

I will enroll the first subject only after all ethical and regulatory requirements are fulfilled. I pledge to obtain written consent for trial participation from all subjects.

I know the requirements for accurate notification of SAEs and I pledge to document and notify such events as described in the protocol.

I pledge to retain all trial-related documents and source data as described. I will provide a curriculum vitae before trial start.

Name (block letters): ______________________________

Function: Investigator

Trial Center (address): ______________________________

______________________________

______________________________

Date: ______________________________

Signature: ______________________________

**Appendices**

Patient information sheet and informed consent form
